# Bone secreted factors induce cellular quiescence in prostate cancer cells

**DOI:** 10.1038/s41598-019-54566-4

**Published:** 2019-12-09

**Authors:** Li-Yuan Yu-Lee, Yu-Chen Lee, Jing Pan, Song-Chang Lin, Tianhong Pan, Guoyu Yu, David H. Hawke, Bih-Fang Pan, Sue-Hwa Lin

**Affiliations:** 1 Department of Medicine, Baylor College of Medicine, Houston, Texas 77030 United States of America; 2 Department of Translational Molecular Pathology, The University of Texas M. D. Anderson Cancer Center, Houston, Texas 77030 United States of America; 3 Department of Orthopedic Oncology, The University of Texas M. D. Anderson Cancer Center, Houston, Texas 77030 United States of America; 4 The Proteomics and Metabolomics Facility, The University of Texas M. D. Anderson Cancer Center, Houston, Texas 77030 United States of America; 5 Department of Genitourinary Medical Oncology, The University of Texas M. D. Anderson Cancer Center, Houston, Texas 77030 United States of America

**Keywords:** Prostate cancer, Cancer microenvironment

## Abstract

Disseminated tumor cells (DTCs) undergo a dormant state in the distant metastatic site(s) before becoming overt metastatic diseases. In prostate cancer (PCa), bone metastasis can occur years after prostatectomy, suggesting that bone may provide dormancy-inducing factors. To search for these factors, we prepared conditioned media (CM) from calvariae. Using live-cell imaging, we found that Calvarial-CM treatment increased cellular quiescence in C4-2B4 PCa cells. Mass spectrometry analysis of Calvarial-CM identified 132 secreted factors. Western blot and ELISA analyses confirmed the presence of several factors, including DKK3, BMP1, neogenin and vasorin in the Calvarial-CM. qRT-PCR analysis of total calvariae versus isolated osteoblasts showed that DKK3, BMP1, vasorin and neogenin are mainly expressed by osteoblasts, while MIA, LECT1, NGAL and PEDF are expressed by other calvarial cells. Recombinant human DKK3, BMP1, vasorin, neogenin, MIA and NGAL treatment increased cellular quiescence in both C4-2b and C4-2B4 PCa cells. Mechanistically, DKK3, vasorin and neogenin, but not BMP1, increased dormancy through activating the p38MAPK signaling pathway. Consistently, DKK3, vasorin and neogenin failed to induce dormancy in cells expressing dominant-negative p38αMAPK while BMP1 remained active, suggesting that BMP1 uses an alternative dormancy signaling pathway. Thus, bone secretes multiple dormancy-inducing factors that employ distinct signaling pathways to induce DTC dormancy in bone.

## Introduction

The majority of men who die of prostate cancer (PCa) have bone metastasis^1^. Bone metastasis can occur years or decades after prostatectomy^2^, suggesting that disseminated tumor cells (DTCs) had been dormant at the metastatic site in bone^3^. Tumor dormancy is now been considered a critical step in the process of metastatic progression during which tumor cells escape therapies that target tumor cells. Dormant tumor cells can become a significant problem at a later time when tumor cells exit dormancy.

Tumor dormancy can be due to instrinsic properties of tumor cells^4^ or due to tumor microenvironment that provides conditions for tumor cells to enter into a quiescent state^3^. In PCa, the bone microenvironment likely plays a critical role in tumor dormancy as bone is the major site of tumor recurrence. How the bone microenvironment promotes DTCs to enter a transient dormant state in bone remains unclear. It is likely that dormancy versus progression of PCa in bone depends on the dynamic interactions between PCa cells and cellular components in bone^5^. Using intravital microscopy, Lawson *et al*.^6^ showed that myeloma cells are dormant when they are in close proximity with the endosteal bone surface in the tibia, but reactivate when they move further away from bone. Wang *et al*.^7^ showed that PCa cells that home to bone are frequently associated with osteoblast lineage cells. Thus, it is likely that the bone microenvironment, especially osteoblasts, are providing dormancy-inducing factors.

Identification of factors in the bone microenvironment that promote tumor dormancy will be an important step in delineating dormancy mechanisms. Two members of the TGFβ/BMP family, GDF10 (BMP-3b) and TGFβ2, secreted from differentiated osteoblasts, have been reported to induce PCa cells to become quiescent *in vitro*^3^. Recently, Wnt5a, an osteoblast secreted factor that maintains hematopoietic stem cells in a quiescent state, was found to also promote PCa cell dormancy in bone^8^. In addition, Shiozawa *et al*.^9,10^ showed that GAS6, a secreted factor that we have also identified in differentiated osteoblasts^3^, also regulates PCa dormancy in the bone marrow. These studies suggest that osteoblasts are one of the cellular components in the bone marrow that support PCa dormancy.

In this study, we looked for additional bone secreted factors that promote cellular dormancy of PCa cells. We used newborn mouse calvariae, which are enriched with osteoblasts^11^, as our study model and identified secreted proteins in the conditioned media. Several of these bone-secreted factors were examined for their effects on inducing cellular quiescence in PCa cells *in vitro* and for their signaling pathway(s) that leads to cellular dormancy.

## Results

### Calvarial conditioned medium (Calvarial-CM) increases cellular quiescence in C4-2B4 PCa cells

**Figure 1:**
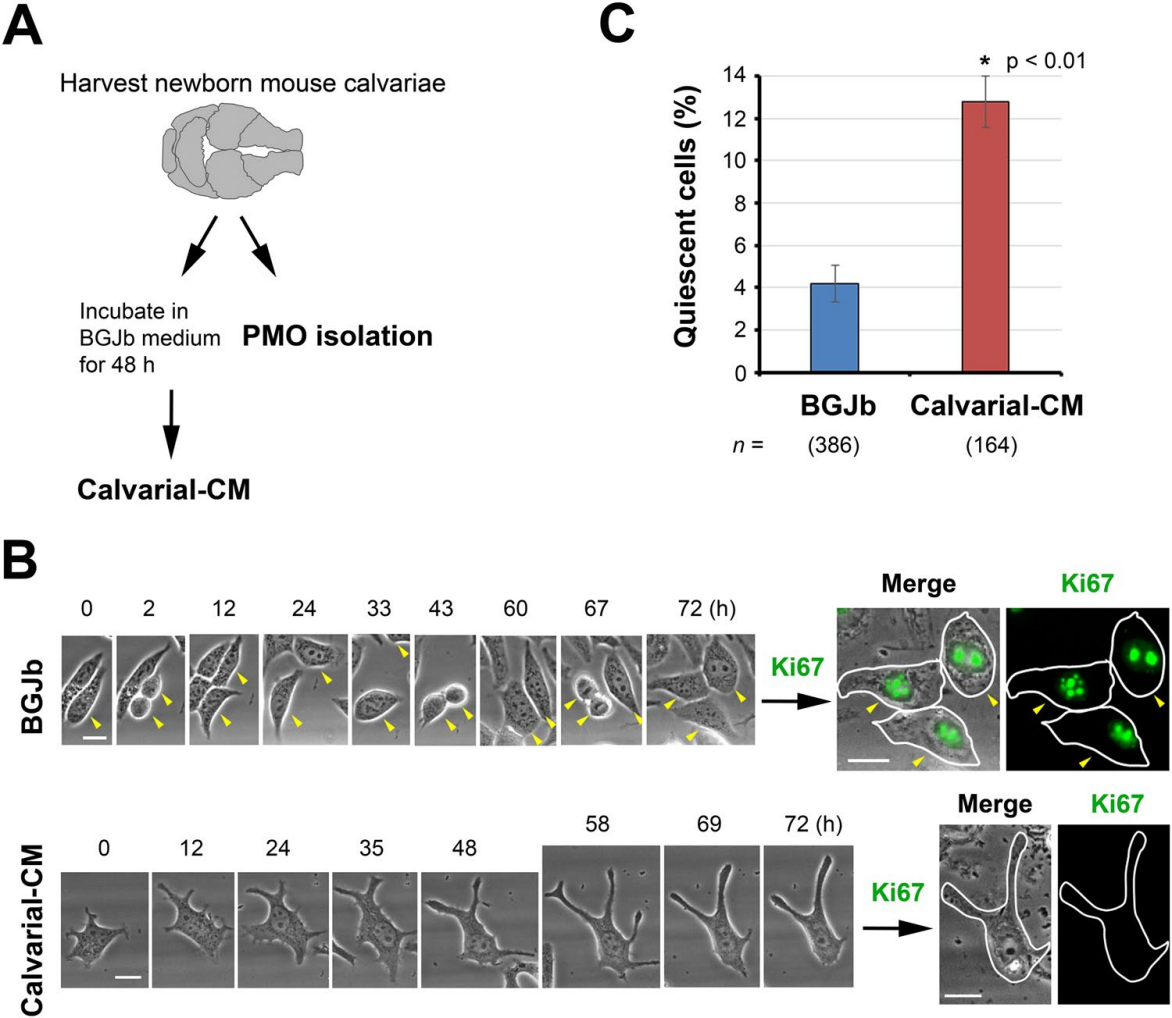
Calvarial conditioned medium (Calvarial-CM) confers cellular quiescence to C4-2B4 PCa cells. (**A**) Calvariae prepared from 2–5 day-old newborn mice were cultured in BGJb medium containing 0.1% BSA for 48 h to generate Calvarial-CM. Calvariae were also used to isolate primary mouse osteoblasts (PMOs) (see details in Materials and Methods). (**B**) Live-cell imaging analysis of C4-2B4 PCa cells incubated in media containing control BGJb media or Calvarial-CM. Single cells were monitored on a Nikon BioStation and images were acquired every 20 min for 72 h. (Left) Phase contrast brightfield images. Arrowheads follow one control cell through three cell divisions. Round cells are undergoing mitosis. Note that one daughter cell left the field of view after T = 33 h. (Right) Immunofluorescence images. Immediately following time-lapse, cells were fixed and immunostained for the proliferation marker Ki67 and re-imaged on the BioStation. Phase contrast images are merged with immunofluorescence images for Ki67. Cell outlines are traced for ease of view. All bars, 20 µm. (**C**) Quantification of % quiescent C4-2B4 cells that did not divide over 72 h relative to total cells examined (mean ± s.e.m.). *n*, number of cells monitored. N, number of independent experiments for control BGJb (N = 5), Calvarial-CM (N = 4). P values were by *t* test.

To identify bone secreted proteins, we used newborn mouse calvariae, which are enriched with osteoblasts^11^. Calvariae prepared from 2–5 day old newborn mice were cultured in BGJb medium containing 0.1% BSA for 48 h to generate calvarial conditioned medium (Calvarial-CM) (Fig. 1A). We have previously shown that this calvarial organ culture condition supports cell proliferation, calvarial bone formation and osteoblast differentiation^12^. To examine whether the Calvarial-CM contains dormancy-inducing activity for PCa cells, C4-2B4 cells were incubated with media containing either control BGJb media or Calvarial-CM and analyzed by live-cell imaging as previously described^3^. Single cells were monitored for cell division over 72 h on a BioStation^3^. While proliferating cells typically undergo 2–3 cell divisions over 72 h under our experimental condition, dormant cells are characterized as viable, non-proliferating or slow-cycling^3,13,14^. In C4-2B4 PCa cells incubated in control media, the vast majority of control cells were observed to undergo several rounds of cell division, as illustrated by following one cell from F0 (T = 0 h) as it rounded up to divide into two F1 progenies (T = 2 h), which flattened out after cell division, to two more cell divisions into F2 (T = 43 h) and then F3 (T = 67 h) progenies (Fig. 1B, arrowheads). In contrast, there was a significant increase in the level of non-proliferating quiescent C4-2B4 cells to 12.8 ± 2.1% when incubated with Calvarial-CM relative to 4.2 ± 1.8% in control BGJb media (Fig. 1C). Immediately following live-cell imaging, cells were stained for the proliferation marker Ki67 and re-imaged on the BioStation. While proliferating cells were positive for Ki67, Calvarial-CM-treated nonproliferating C4-2B4 cells were Ki67 negative (Fig. 1B, right). These observations suggest that the Calvarial-CM contains factors that induced cellular quiescence of C4-2B4 cells.

### Secretome analysis of bone conditioned medium (Bone-CM)

**Figure 2:**
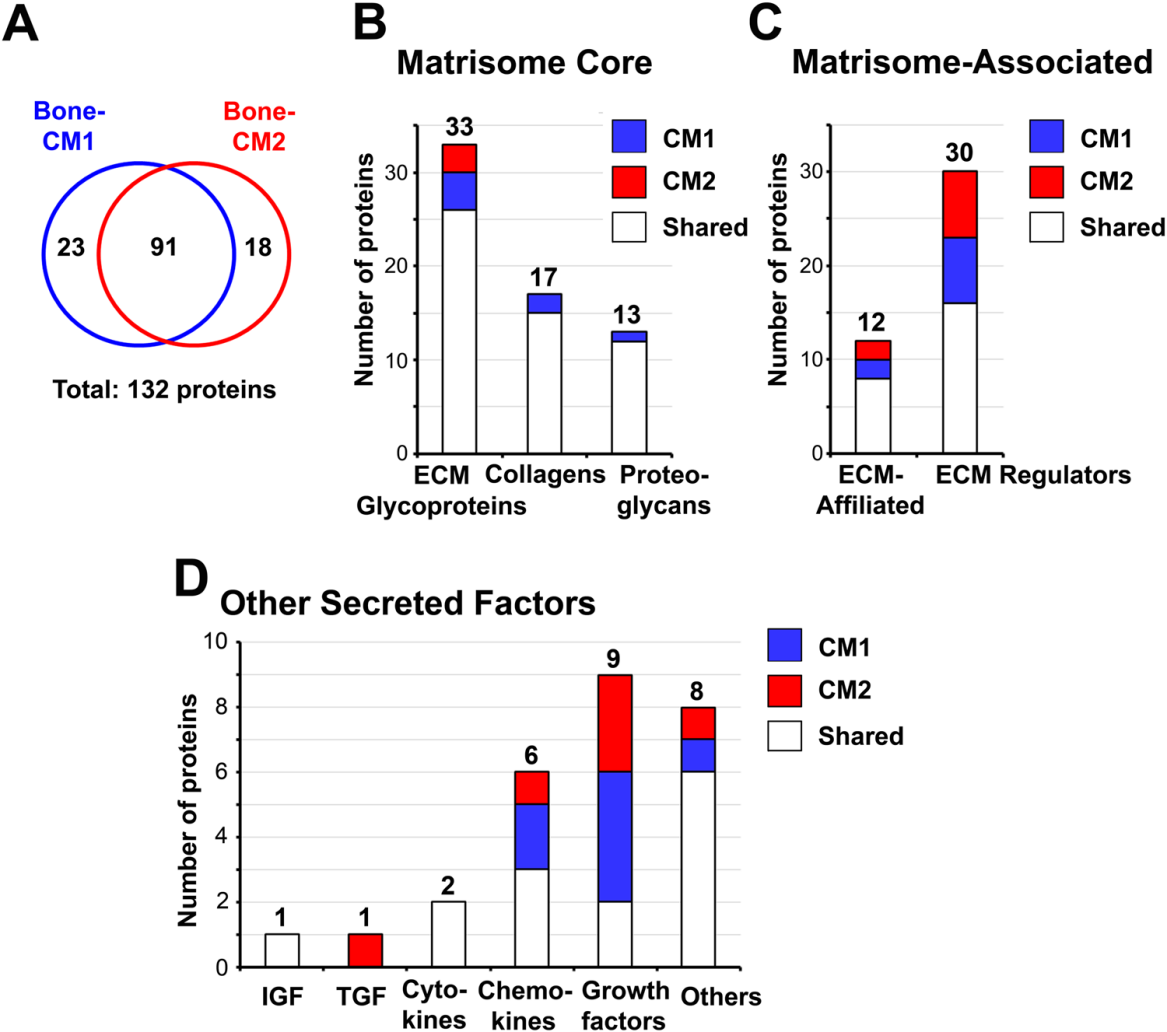
Proteomics analysis of proteins from bone conditioned media. (**A**) Venn diagram of secreted proteins identified in Bone-CM1 versus Bone-CM2. Two independent batches of Bone-CM were subjected to LC-MS/MS analysis. Proteins were selected using 1% false discovery rate (FDR). We focused on the secreted proteins and obtained a total of 132 secreted proteins from the Bone-CM. We grouped the 132 secreted proteins into three major categories of extracellular matrix (ECM) proteins according to the Matrisome Project (http://matrisomeproject.mit.edu)^15^. We further curated our list of secreted proteins by searching against the matrisome atlas using the MatrisomeDB2.0 software^15,16^. (**B**) Matrisome core proteins include ECM-glycoportiens, collagens and proteoglycans. (**C**) Matrisome-associated proteins include ECM-affiliated proteins and ECM regulators. (**D**) Other secreted factors include growth factors and other proteins. For a complete list of proteins identified in Bone-CM1 (n = 416) and Bone-CM2 (n = 244), see Supplemental Tables S1 and S2.

To identify potential dormancy-inducing factors secreted from calvariae, two independent calvarial preparations cultured in BSA-free medium, referred to as Bone-CM1 and Bone-CM2, to distinguish from Calvarial-CM that contained BSA, were concentrated 20-fold and analyzed by LC-MS/MS. Using a false discovery rate (FDR) of 1%, 416 and 244 proteins were identified from Bone-CM1 (Supplemental Table S1) and Bone-CM2 (Supplemental Table S2), respectively. Among these proteins, 114 and 109 proteins are secreted proteins from Bone-CM1 and Bone-CM2, respectively, based on UniProt mouse database. Using the UniProt database, we identified factors that are known to be secreted proteins and additional factors belonging to type I single-pass transmembrane proteins whose extracellular domain can be processed and released as a soluble fragment into the extracellular space. In this manner, 91 proteins were found in both samples, while 23 proteins were additionally found only in Bone-CM1 and 18 only in Bone-CM2 (Fig. 2A). Thus, a total of 132 secreted proteins were identified in the Bone-CM (Table 1).

**Table 1:** Secretome of mouse calvariae.

Protein Accession	Gene Name	Protein Description	MW [kDa]	Protein Score	# Signif. Matches	# Unique Peptides	Sequence Coverage	Bone-CM1*	Bone-CM2*
**Other Secreted Factors**									
**IGF family**									
[IGF2_MOUSE]	Igf2	Insulin-like growth factor II OS = Mus musculus GN = Igf2 PE = 1 SV = 1	20.0	113.83	4	2	12.78	yes	yes
**TGF family**									
[TGFB1_MOUSE]	Tgfb1	Transforming growth factor beta-1 OS = Mus musculus GN = Tgfb1 PE = 1 SV = 1	44.3	31.51	1	1	5.13		yes
**Growth Factors**									
[FETUA_MOUSE]	Ahsg	Alpha-2-HS-glycoprotein OS = Mus musculus GN = Ahsg PE = 1 SV = 1	37.3	252.56	7	4	15.94	yes	
[DKK3_MOUSE]	Dkk3	Dickkopf-related protein 3 OS = Mus musculus GN = Dkk3 PE = 2 SV = 1	38.4	136.07	2	2	10.60		yes
[NGAL_MOUSE]	Lcn2	Neutrophil gelatinase-associated lipocalin OS = Mus musculus GN = Lcn2 PE = 1 SV = 1	22.9	128.01	4	3	16.50		yes
[FSTL1_MOUSE]	Fstl1	Follistatin-related protein 1 OS = Mus musculus GN = Fstl1 PE = 1 SV = 2	34.5	113.85	3	3	11.76	yes	yes
[NPTX1_MOUSE]	Nptx1	Neuronal pentraxin-1 OS = Mus musculus GN = Nptx1 PE = 2 SV = 1	47.1	68.33	1	1	5.09		yes
[VASN_MOUSE]	Vasn	Vasorin OS = Mus musculus GN = Vasn PE = 2 SV = 2	72.2	65.32	2	2	7.88	yes	
[MIA_MOUSE]	Mia	Melanoma-derived growth regulatory protein OS = Mus musculus GN = Mia PE = 2 SV = 2	14.6	34.91	1	1	6.92	yes	yes
[NEO1_MOUSE]	Neo1	Neogenin OS = Mus musculus GN = Neo1 PE = 1 SV = 1	163.1	31.17	1	1	1.21	yes	
[LECT1_MOUSE]	Lect1	Leukocyte cell-derived chemotaxin 1 OS = Mus musculus GN = Lect1 PE = 1 SV = 2	37.2	26.23	1	1	2.10	yes	
**Cytokines**									
[CSF1_MOUSE]	Csf1	Macrophage colony-stimulating factor 1 OS = Mus musculus GN = Csf1 PE = 1 SV = 2	60.6	72.58	1	1	2.54	yes	yes
[MIF_MOUSE]	Mif	Macrophage migration inhibitory factor OS = Mus musculus GN = Mif PE = 1 SV = 2	12.5	71.44	1	1	9.57	yes	yes
**Chemokines**									
[S10A9_MOUSE]	S100a9	Protein S100-A9 OS = Mus musculus GN = S100a9 PE = 1 SV = 3	13.0	184.25	8	4	38.94	yes	
[S10AA_MOUSE]	S100a10	Protein S100-A10 OS = Mus musculus GN = S100a10 PE = 1 SV = 2	11.2	163.77	6	3	49.48	yes	yes
[S10A8_MOUSE]	S100a8	Protein S100-A8 OS = Mus musculus GN = S100a8 PE = 1 SV = 3	10.3	140.97	7	2	50.56	yes	yes
[S10AD_MOUSE]	S100a13	Protein S100-A13 OS = Mus musculus GN = S100a13 PE = 1 SV = 1	11.2	96.16	2	2	23.47	yes	
[S10AB_MOUSE]	S100a11	Protein S100-A11 OS = Mus musculus GN = S100a11 PE = 1 SV = 1	11.1	74.64	3	1	16.33	yes	yes
[S10A6_MOUSE]	S100a6	Protein S100-A6 OS = Mus musculus GN = S100a6 PE = 1 SV = 3	10.0	46.38	1	1	8.99		yes
**Others**									
[NUCB1_MOUSE]	Nucb1	Nucleobindin-1 OS = Mus musculus GN = Nucb1 PE = 1 SV = 2	53.4	506.84	12	9	24.84	yes	yes
[CALU_MOUSE]	Calu	Calumenin OS = Mus musculus GN = Calu PE = 1 SV = 1	37.0	337.55	7	6	31.75	yes	yes
[CH3L1_MOUSE]	Chi3l1	Chitinase-3-like protein 1 OS = Mus musculus GN = Chi3l1 PE = 1 SV = 3	43.9	327.84	11	6	21.08		yes
[PPBT_MOUSE]	Alpl	Alkaline phosphatase OS = Mus musculus GN = Alpl PE = 1 SV = 2	57.5	299.32	6	5	15.27	yes	yes
[CLUS_MOUSE]	Clu	Clusterin OS = Mus musculus GN = Clu PE = 1 SV = 1	51.6	193.06	5	4	10.94		yes
[OLFL3_MOUSE]	Olfml3	Olfactomedin-like protein 3 OS = Mus musculus GN = Olfml3 PE = 2 SV = 2	45.7	158.37	3	3	7.88	yes	yes
[NGP_MOUSE]	Ngp	Neutrophilic granule protein OS = Mus musculus GN = Ngp PE = 1 SV = 1	19.3	83.71	1	1	12.57	yes	
[UCMA_MOUSE]	Ucma	Unique cartilage matrix-associated protein OS = Mus musculus GN = Ucma PE = 1 SV = 1	16.6	76.49	1	1	9.42	yes	yes
[SAP_MOUSE]	Psap	Prosaposin OS = Mus musculus GN = Psap PE = 1 SV = 2	61.4	69.91	3	1	3.59	yes	yes
[CCD80_MOUSE]	Ccdc80	Coiled-coil domain-containing protein 80 OS = Mus musculus GN = Ccdc80 PE = 1 SV = 2	107.5	60.21	1	1	1.26	yes	
[DAG1_MOUSE]	Dag1	Dystroglycan OS = Mus musculus GN = Dag1 PE = 1 SV = 4	96.8	57.62	1	1	1.79		yes
[CRTAP_MOUSE]	Crtap	Cartilage-associated protein OS = Mus musculus GN = Crtap PE = 1 SV = 3	46.1	51.66	1	1	2.75	yes	
[CDSN_MOUSE]	Cdsn	Corneodesmosin OS = Mus musculus GN = Cdsn PE = 2 SV = 2	54.3	36.81	1	1	3.21		yes
**Matrisome Core Proteins**									
**ECM-Glycoproteins**									
[FINC_MOUSE]	Fn1	Fibronectin OS = Mus musculus GN = Fn1 PE = 1 SV = 4	272.4	3888.07	171	66	40.09	yes	yes
[TENA_MOUSE]	Tnc	Tenascin OS = Mus musculus GN = Tnc PE = 1 SV = 1	231.7	2310.24	56	39	27.20	yes	yes
[TENN_MOUSE]	Tnn	Tenascin-N OS = Mus musculus GN = Tnn PE = 1 SV = 2	173.0	2200.77	61	37	38.40	yes	yes
[POSTN_MOUSE]	Postn	Periostin OS = Mus musculus GN = Postn PE = 1 SV = 2	93.1	1000.50	33	19	30.43	yes	yes
[BGH3_MOUSE]	Tgfbi	Transforming growth factor-beta-induced protein ig-h3 OS = Mus musculus GN = Tgfbi PE = 1 SV = 1	74.5	703.94	25	12	23.87	yes	yes
[OSTP_MOUSE]	Spp1	Osteopontin OS = Mus musculus GN = Spp1 PE = 1 SV = 1	32.4	669.32	19	10	44.90	yes	yes
[AEBP1_MOUSE]	Aebp1	Adipocyte enhancer-binding protein 1 OS = Mus musculus GN = Aebp1 PE = 1 SV = 1	128.3	435.94	7	7	9.40	yes	yes
[TSP1_MOUSE]	Thbs1	Thrombospondin-1 OS = Mus musculus GN = Thbs1 PE = 1 SV = 1	129.6	390.40	12	8	9.66	yes	yes
[PCOC1_MOUSE]	Pcolce	Procollagen C-endopeptidase enhancer 1 OS = Mus musculus GN = Pcolce PE = 1 SV = 2	50.1	388.92	9	7	22.44	yes	yes
[MATN4_MOUSE]	Matn4	Matrilin-4 OS = Mus musculus GN = Matn4 PE = 2 SV = 1	68.9	375.75	13	7	21.96	yes	yes
[MATN1_MOUSE]	Matn1	Cartilage matrix protein (Matrilin-1) OS = Mus musculus GN = Matn1 PE = 2 SV = 2	54.4	372.12	6	6	19.40	yes	yes
[EMIL1_MOUSE]	Emilin1	EMILIN-1 OS = Mus musculus GN = Emilin1 PE = 1 SV = 1	107.5	294.76	5	4	7.87	yes	yes
[LAMB1_MOUSE]	Lamb1	Laminin subunit beta-1 OS = Mus musculus GN = Lamb1 PE = 1 SV = 3	197.0	281.37	4	3	3.47	yes	yes
[SPRC_MOUSE]	Sparc	SPARC OS = Mus musculus GN = Sparc PE = 1 SV = 1	34.4	280.06	11	5	16.23	yes	yes
[NID2_MOUSE]	Nid2	Nidogen-2 OS = Mus musculus GN = Nid2 PE = 1 SV = 2	153.8	233.27	5	4	5.20	yes	yes
[COMP_MOUSE]	Comp	Cartilage oligomeric matrix protein OS = Mus musculus GN = Comp PE = 1 SV = 2	82.3	203.03	4	4	7.55	yes	yes
[TSP2_MOUSE]	Thbs2	Thrombospondin-2 OS = Mus musculus GN = Thbs2 PE = 1 SV = 2	129.8	168.15	3	3	3.67	yes	yes
[LAMA4_MOUSE]	Lama4	Laminin subunit alpha-4 OS = Mus musculus GN = Lama4 PE = 1 SV = 2	201.7	160.30	3	3	2.92	yes	yes
[NID1_MOUSE]	Nid1	Nidogen-1 OS = Mus musculus GN = Nid1 PE = 1 SV = 2	136.5	151.10	3	3	5.46	yes	yes
[FBLN5_MOUSE]	Fbln5	Fibulin-5 OS = Mus musculus GN = Fbln5 PE = 1 SV = 1	50.2	127.36	3	3	9.15	yes	yes
[FBLN2_MOUSE]	Fbln2	Fibulin-2 OS = Mus musculus GN = Fbln2 PE = 1 SV = 2	131.7	103.63	2	2	2.29	yes	yes
[FBLN1_MOUSE]	Fbln1	Fibulin-1 OS = Mus musculus GN = Fbln1 PE = 1 SV = 2	78.0	95.22	3	2	4.96	yes	yes
[IBP5_MOUSE]	Igfbp5	Insulin-like growth factor-binding protein 5 OS = Mus musculus GN = Igfbp5 PE = 1 SV = 1	30.4	92.91	2	2	9.59	yes	yes
[FBLN4_MOUSE]	Efemp2	EGF-containing fibulin-like extracellular matrix protein 2 OS = Mus musculus GN = Efemp2 PE = 1 SV = 1	49.4	88.66	3	2	7.67	yes	yes
[MATN2_MOUSE]	Matn2	Matrilin-2 OS = Mus musculus GN = Matn2 PE = 2 SV = 2	106.7	80.75	1	1	2.09	yes	
[LAMC1_MOUSE]	Lamc1	Laminin subunit gamma-1 OS = Mus musculus GN = Lamc1 PE = 1 SV = 2	177.2	77.95	1	1	1.06	yes	yes
[MATN3_MOUSE]	Matn3	Matrilin-3 OS = Mus musculus GN = Matn3 PE = 1 SV = 2	51.8	76.56	1	1	6.03	yes	yes
[HMCN1_MOUSE]	Hmcn1	Hemicentin-1 (fibulin 6) OS = Mus musculus GN = Hmcn1 PE = 1 SV = 1	611.2	63.31	1	1	0.28	yes	
[IBP7_MOUSE]	Igfbp7	Insulin-like growth factor-binding protein 7 OS = Mus musculus GN = Igfbp7 PE = 1 SV = 3	29.0	59.65	1	1	4.63		yes
[VITRN_MOUSE]	Vit	Vitrin OS = Mus musculus GN = Vit PE = 1 SV = 2	70.7	55.83	2	1	5.38	yes	
[SMOC2_MOUSE]	Smoc2	SPARC-related modular calcium-binding protein 2 OS = Mus musculus GN = Smoc2 PE = 1 SV = 1	49.9	43.80	1	1	1.79		yes
**Collagens**									
[COCA1_MOUSE]	Col12a1	Collagen alpha-1(XII) chain OS = Mus musculus GN = Col12a1 PE = 2 SV = 3	340.0	4822.72	137	82	37.88	yes	yes
[CO1A2_MOUSE]	Col1a2	Collagen alpha-2(I) chain OS = Mus musculus GN = Col1a2 PE = 2 SV = 2	129.5	2264.87	56	38	48.54	yes	yes
[CO1A1_MOUSE]	Col1a1	Collagen alpha-1(I) chain OS = Mus musculus GN = Col1a1 PE = 1 SV = 4	137.9	2013.20	51	38	43.15	yes	yes
[CO2A1_MOUSE]	Col2a1	Collagen alpha-1(II) chain OS = Mus musculus GN = Col2a1 PE = 1 SV = 2	141.9	1997.23	53	36	34.50	yes	yes
[COBA1_MOUSE]	Col11a1	Collagen alpha-1(XI) chain OS = Mus musculus GN = Col11a1 PE = 1 SV = 2	180.9	914.89	33	14	13.91	yes	yes
[CO6A1_MOUSE]	Col6a1	Collagen alpha-1(VI) chain OS = Mus musculus GN = Col6a1 PE = 2 SV = 1	108.4	759.85	18	11	15.61	yes	yes
[COBA2_MOUSE]	Col11a2	Collagen alpha-2(XI) chain OS = Mus musculus GN = Col11a2 PE = 2 SV = 3	171.4	534.18	27	12	9.74	yes	yes
[CO9A1_MOUSE]	Col9a1	Collagen alpha-1(IX) chain OS = Mus musculus GN = Col9a1 PE = 2 SV = 2	92.0	497.54	12	8	12.27	yes	yes
[COEA1_MOUSE]	Col14a1	Collagen alpha-1(XIV) chain OS = Mus musculus GN = Col14a1 PE = 2 SV = 2	192.9	448.31	15	8	7.29	yes	yes
[CO5A1_MOUSE]	Col5a1	Collagen alpha-1(V) chain OS = Mus musculus GN = Col5a1 PE = 2 SV = 2	183.6	424.06	12	7	8.60	yes	yes
[CO6A2_MOUSE]	Col6a2	Collagen alpha-2(VI) chain OS = Mus musculus GN = Col6a2 PE = 2 SV = 3	110.3	306.96	7	7	8.22	yes	yes
[CO9A2_MOUSE]	Col9a2	Collagen alpha-2(IX) chain OS = Mus musculus GN = Col9a2 PE = 2 SV = 1	65.3	263.90	5	4	8.14	yes	yes
[CO3A1_MOUSE]	Col3a1	Collagen alpha-1(III) chain OS = Mus musculus GN = Col3a1 PE = 1 SV = 4	138.9	211.05	5	5	5.46	yes	yes
[CO4A1_MOUSE]	Col4a1	Collagen alpha-1(IV) chain OS = Mus musculus GN = Col4a1 PE = 1 SV = 4	160.6	61.42	4	1	1.86	yes	
[CO5A2_MOUSE]	Col5a2	Collagen alpha-2(V) chain OS = Mus musculus GN = Col5a2 PE = 1 SV = 1	144.9	58.70	3	1	1.47	yes	yes
[COIA1_MOUSE]	Col18a1	Collagen alpha-1(XVIII) chain OS = Mus musculus GN = Col18a1 PE = 1 SV = 4	182.1	48.25	1	1	1.86	yes	
[CO6A5_MOUSE]	Col6a5	Collagen alpha-5(VI) chain OS = Mus musculus GN = Col6a5 PE = 1 SV = 4	289.4	31.88	1	1	0.45	yes	yes
**Proteoglycans**									
[PGBM_MOUSE]	Hspg2	Basement membrane-specific heparan sulfate proteoglycan core protein (Perlecan) OS = Mus musculus GN = Hspg2 PE = 1 SV = 1	398.0	2254.48	58	42	17.94	yes	yes
[LUM_MOUSE]	Lum	Lumican OS = Mus musculus GN = Lum PE = 1 SV = 2	38.2	623.55	24	11	31.36	yes	yes
[PGS1_MOUSE]	Bgn	Biglycan OS = Mus musculus GN = Bgn PE = 1 SV = 1	41.6	493.16	21	8	24.93	yes	yes
[PGCA_MOUSE]	Acan	Aggrecan core protein OS = Mus musculus GN = Acan PE = 1 SV = 2	221.8	453.63	16	10	5.58	yes	yes
[MIME_MOUSE]	Ogn	Mimecan OS = Mus musculus GN = Ogn PE = 1 SV = 1	34.0	401.06	14	6	19.80	yes	yes
[HPLN1_MOUSE]	Hapln1	Hyaluronan and proteoglycan link protein 1 OS = Mus musculus GN = Hapln1 PE = 1 SV = 1	40.5	295.58	7	6	18.26	yes	yes
[PGS2_MOUSE]	Dcn	Decorin OS = Mus musculus GN = Dcn PE = 1 SV = 1	39.8	232.66	6	5	14.97	yes	yes
[CSPG2_MOUSE]	Vcan	Versican core protein OS = Mus musculus GN = Vcan PE = 1 SV = 2	366.6	212.58	5	3	1.64	yes	yes
[FMOD_MOUSE]	Fmod	Fibromodulin OS = Mus musculus GN = Fmod PE = 2 SV = 1	43.0	183.28	4	4	17.29	yes	yes
[EPYC_MOUSE]	Epyc	Epiphycan OS = Mus musculus GN = Epyc PE = 2 SV = 1	36.7	95.30	1	1	5.28	yes	yes
[OMD_MOUSE]	Omd	Osteomodulin OS = Mus musculus GN = Omd PE = 2 SV = 1	49.7	77.73	2	2	7.80	yes	
[CHADL_MOUSE]	Chadl	Chondroadherin-like protein OS = Mus musculus GN = Chadl PE = 1 SV = 1	81.3	66.49	1	1	2.27	yes	yes
[PRG4_MOUSE]	Prg4	Proteoglycan 4 OS = Mus musculus GN = Prg4 PE = 1 SV = 2	115.9	58.62	1	1	1.52	yes	yes
**Matrisome-Associated**									
**ECM-Affiliated**									
[CSPG4_MOUSE]	Cspg4	Chondroitin sulfate proteoglycan 4 OS = Mus musculus GN = Cspg4 PE = 1 SV = 3	252.2	788.32	23	14	10.27	yes	yes
[ANXA2_MOUSE]	Anxa2	Annexin A2 OS = Mus musculus GN = Anxa2 PE = 1 SV = 2	38.7	659.62	16	11	43.07	yes	yes
[ANXA5_MOUSE]	Anxa5	Annexin A5 OS = Mus musculus GN = Anxa5 PE = 1 SV = 1	35.7	488.72	12	9	28.53	yes	yes
[ANXA6_MOUSE]	Anxa6	Annexin A6 OS = Mus musculus GN = Anxa6 PE = 1 SV = 3	75.8	274.22	4	4	9.36	yes	
[LEG1_MOUSE]	Lgals1	Galectin-1 OS = Mus musculus GN = Lgals1 PE = 1 SV = 3	14.9	266.61	17	5	34.07	yes	yes
[GPC1_MOUSE]	Gpc1	Glypican-1 OS = Mus musculus GN = Gpc1 PE = 1 SV = 1	61.3	238.48	3	3	11.31		yes
[ANXA1_MOUSE]	Anxa1	Annexin A1 OS = Mus musculus GN = Anxa1 PE = 1 SV = 2	38.7	222.80	5	4	15.32	yes	yes
[CLC11_MOUSE]	Clec11a	C-type lectin domain family 11 member A OS = Mus musculus GN = Clec11a PE = 2 SV = 1	36.4	123.68	2	2	9.45	yes	yes
[TETN_MOUSE]	Clec3b	Tetranectin OS = Mus musculus GN = Clec3b PE = 1 SV = 2	22.2	67.39	2	1	5.94	yes	
[CLC3A_MOUSE]	Clec3a	C-type lectin domain family 3 member A OS = Mus musculus GN = Clec3a PE = 3 SV = 1	22.2	41.91	1	1	10.20	yes	yes
[LEG3_MOUSE]	Lgals3	Galectin-3 OS = Mus musculus GN = Lgals3 PE = 1 SV = 3	27.5	35.01	1	1	4.92	yes	yes
[SEM7A_MOUSE]	Sema7a	Semaphorin-7A OS = Mus musculus GN = Sema7a PE = 1 SV = 1	74.9	32.36	1	1	2.26		yes
**ECM Regulators**									
[SERPH_MOUSE]	Serpinh1	Serpin H1 OS = Mus musculus GN = Serpinh1 PE = 1 SV = 3	46.5	1089.19	43	17	51.80	yes	yes
[MMP13_MOUSE]	Mmp13	Collagenase 3 OS = Mus musculus GN = Mmp13 PE = 1 SV = 1	54.1	1082.35	29	19	56.14	yes	yes
[PEDF_MOUSE]	Serpinf1	Pigment epithelium-derived factor OS = Mus musculus GN = Serpinf1 PE = 1 SV = 2	46.2	1009.46	36	17	49.88	yes	yes
[MMP2_MOUSE]	Mmp2	72 kDa type IV collagenase OS = Mus musculus GN = Mmp2 PE = 2 SV = 1	74.1	432.22	11	8	25.83	yes	yes
[CD109_MOUSE]	Cd109	CD109 antigen OS = Mus musculus GN = Cd109 PE = 2 SV = 1	161.6	416.76	7	7	7.98	yes	
[MMP3_MOUSE]	Mmp3	Stromelysin-1 OS = Mus musculus GN = Mmp3 PE = 2 SV = 2	53.8	401.98	11	10	25.16	yes	yes
[SPA3N_MOUSE]	Serpina3n	Serine protease inhibitor A3N OS = Mus musculus GN = Serpina3n PE = 1 SV = 1	46.7	252.13	5	4	14.83	yes	yes
[CYTC_MOUSE]	Cst3	Cystatin-C OS = Mus musculus GN = Cst3 PE = 2 SV = 2	15.5	213.65	5	4	37.86	yes	yes
[CATS_MOUSE]	Ctss	Cathepsin S OS = Mus musculus GN = Ctss PE = 1 SV = 2	38.4	204.21	4	4	13.24		yes
[CATB_MOUSE]	Ctsb	Cathepsin B OS = Mus musculus GN = Ctsb PE = 1 SV = 2	37.3	170.41	4	3	14.16	yes	yes
[ITIH1_MOUSE]	Itih1	Inter-alpha-trypsin inhibitor heavy chain H1 OS = Mus musculus GN = Itih1 PE = 1 SV = 2	101.0	136.04	3	3	11.03	yes	
[SPA3C_MOUSE]	Serpina3c	Serine protease inhibitor A3C OS = Mus musculus GN = Serpina3c PE = 2 SV = 1	46.7	131.55	5	1	9.35	yes	yes
[CATD_MOUSE]	Ctsd	Cathepsin D OS = Mus musculus GN = Ctsd PE = 1 SV = 1	44.9	125.57	3	2	10.49	yes	yes
[BMP1_MOUSE]	Bmp1	Bone morphogenetic protein 1 OS = Mus musculus GN = Bmp1 PE = 1 SV = 2	111.6	112.07	1	1	1.61		yes
[ITIH2_MOUSE]	Itih2	Inter-alpha-trypsin inhibitor heavy chain H2 OS = Mus musculus GN = Itih2 PE = 1 SV = 1	105.9	110.29	2	2	4.97	yes	
[GDN_MOUSE]	Serpine2	Glia-derived nexin (serpin E) OS = Mus musculus GN = Serpine2 PE = 1 SV = 2	44.2	106.05	2	2	8.06		yes
[A2MP_MOUSE]	A2mp	Alpha-2-macroglobulin-P OS = Mus musculus GN = A2mp PE = 2 SV = 2	164.2	80.38	1	1	1.49	yes	yes
[CATL1_MOUSE]	Ctsl	Cathepsin L1 OS = Mus musculus GN = Ctsl PE = 1 SV = 2	37.5	77.02	2	2	6.59	yes	yes
[ADA15_MOUSE]	Adam15	Disintegrin and metalloproteinase domain-containing protein 15 OS = Mus musculus GN = Adam15 PE = 1 SV = 2	92.6	71.85	1	1	2.66		yes
[PAI2_MOUSE]	Serpinb2	Plasminogen activator inhibitor 2, macrophage OS = Mus musculus GN = Serpinb2 PE = 2 SV = 1	46.3	64.13	1	1	3.37		yes
[TIMP1_MOUSE]	Timp1	Metalloproteinase inhibitor 1 OS = Mus musculus GN = Timp1 PE = 1 SV = 2	22.6	56.58	1	1	4.39	yes	yes
[MMP12_MOUSE]	Mmp12	Macrophage metalloelastase OS = Mus musculus GN = Mmp12 PE = 1 SV = 3	54.9	46.86	1	1	2.54		yes
[TIMP2_MOUSE]	Timp2	Metalloproteinase inhibitor 2 OS = Mus musculus GN = Timp2 PE = 1 SV = 2	24.3	41.30	2	1	6.82	yes	yes
[ITIH3_MOUSE]	Itih3	Inter-alpha-trypsin inhibitor heavy chain H3 OS = Mus musculus GN = Itih3 PE = 1 SV = 3	99.3	32.43	1	1	1.01	yes	
[LYOX_MOUSE]	Lox	Protein-lysine 6-oxidase OS = Mus musculus GN = Lox PE = 1 SV = 1	46.7	30.68	1	1	2.68	yes	yes
[LOXL3_MOUSE]	Loxl3	Lysyl oxidase homolog 3 OS = Mus musculus GN = Loxl3 PE = 2 SV = 2	83.7	29.26	1	1	1.46	yes	
[PAI1_MOUSE]	Serpine1	Plasminogen activator inhibitor 1 OS = Mus musculus GN = Serpine1 PE = 1 SV = 1	45.1	27.19	1	1	7.71	yes	

*Identified in bone-conditioned media Bone-CM1, Bone-CM2, or both as indicated.

### Analysis of secreted proteins in bone conditioned medium (Bone-CM)

Next, we compared our list of bone-secreted proteins to the extracellular matrix (ECM) protein atlas compiled by the Matrisome Project (http://matrisomeproject.mit.edu)^15,16^. The ECM protein atlas contains an inventory of matrisomes identified from normal lung, liver and colon tissues as well as metastatic colon, melanoma, myeloma and mammary tissues^15–18^, ovary^19^, endothelial cells^20^, pancreatic islet cells^21^, and various stem-cell derived ECMs^22^. According to the classification of the matrisome proteome^15^, the 132 bone-secreted proteins can be grouped into three major categories: “Matrisome Core” proteins (63 proteins), “Matrisome-Associated” proteins (42 proteins), and “Other Secreted Factors” (27 proteins). The 63 proteins that belong to the “Matrisome Core” are subgrouped to ECM glycoproteins, collagens and proteoglycans (Fig. 2B). The 42 proteins that belong to the “Matrisome-Associated” category are subgrouped to ECM-affiliated proteins and ECM regulator proteins (Fig. 2C). The rest of the other secreted factors are subgrouped to the IGF, TGF, cytokine, chemokine and growth factor families (Fig. 2D).

Among the 27 other secreted factors, we found that 21 of the bone-secreted proteins have not been identified in other matrisomes. These 21 proteins are listed in Table 2 and include 8 growth factors and 13 other types of secreted factors. Among the growth factors are Dickkopf-related protein 3 (DKK3)^23^, also known as a tumor suppressor (“reduced expression in carcinoma REIC”)^24–26^; melanoma-derived growth regulatory protein (MIA)^27^, a factor that inhibits melanoma cell growth and is also known as “cartilage-derived retinoic acid-sensitive protein (CD-RAP)” with a role in cartilage differentiation^28^; and neutrophil gelatinase-associated lipocalin (NGAL)^29^, a factor that promotes renal epithelial cell differentiation. Neogenin (NEO1), a protein that can bind and modulate BMP signaling^30,31^ and is involved in the transition of proliferating cells to their differentiated state^32,33^; leukocyte cell-derived chemotaxin-1 (LECT1), also known as chrondromodulin, a protein involved in inhibiting the growth of vascular endothelial cells and tumor cells^34^; and vasorin (VASN), a protein that binds to and antagonizes TGFβ1 signaling^35^, are also found in the list of growth factors (Table 2). Together, these factors are potential candidates in the bone secretome that may play a role in regulating PCa activity in bone.

**Table 2:** Secreted factors unique in bone-conditioned media.

Protein Accession	Gene Name	Protein Description	MW [kDa]	Protein Score	# Signif. Matches	# Unique Peptides	Sequence Coverage	Bone-CM1*	Bone-CM2*
**Growth Factors**									
[FETUA_MOUSE]	Ahsg	Alpha-2-HS-glycoprotein OS = Mus musculus GN = Ahsg PE = 1 SV = 1	37.3	252.56	7	4	15.94	yes	
[DKK3_MOUSE]	Dkk3	Dickkopf-related protein 3 OS = Mus musculus GN = Dkk3 PE = 2 SV = 1	38.4	136.07	2	2	10.60		yes
[NGAL_MOUSE]	Lcn2	Neutrophil gelatinase-associated lipocalin OS = Mus musculus GN = Lcn2 PE = 1 SV = 1	22.9	128.01	4	3	16.50		yes
[NPTX1_MOUSE]	Nptx1	Neuronal pentraxin-1 OS = Mus musculus GN = Nptx1 PE = 2 SV = 1	47.1	68.33	1	1	5.09		yes
[VASN_MOUSE]	Vasn	Vasorin OS = Mus musculus GN = Vasn PE = 2 SV = 2	72.2	65.32	2	2	7.88	yes	
[MIA_MOUSE]	Mia	Melanoma-derived growth regulatory protein OS = Mus musculus GN = Mia PE = 2 SV = 2	14.6	34.91	1	1	6.92	yes	yes
[NEO1_MOUSE]	Neo1	Neogenin OS = Mus musculus GN = Neo1 PE = 1 SV = 1	163.1	31.17	1	1	1.21	yes	
[LECT1_MOUSE]	Lect1	Leukocyte cell-derived chemotaxin 1 OS = Mus musculus GN = Lect1 PE = 1 SV = 2	37.2	26.23	1	1	2.10	yes	
**Others**									
[NUCB1_MOUSE]	Nucb1	Nucleobindin-1 OS = Mus musculus GN = Nucb1 PE = 1 SV = 2	53.4	506.84	12	9	24.84	yes	yes
[CALU_MOUSE]	Calu	Calumenin OS = Mus musculus GN = Calu PE = 1 SV = 1	37.0	337.55	7	6	31.75	yes	yes
[CH3L1_MOUSE]	Chi3l1	Chitinase-3-like protein 1 OS = Mus musculus GN = Chi3l1 PE = 1 SV = 3	43.9	327.84	11	6	21.08		yes
[PPBT_MOUSE]	Alpl	Alkaline phosphatase OS = Mus musculus GN = Alpl PE = 1 SV = 2	57.5	299.32	6	5	15.27	yes	yes
[CLUS_MOUSE]	Clu	Clusterin OS = Mus musculus GN = Clu PE = 1 SV = 1	51.6	193.06	5	4	10.94		yes
[OLFL3_MOUSE]	Olfml3	Olfactomedin-like protein 3 OS = Mus musculus GN = Olfml3 PE = 2 SV = 2	45.7	158.37	3	3	7.88	yes	yes
[NGP_MOUSE]	Ngp	Neutrophilic granule protein OS = Mus musculus GN = Ngp PE = 1 SV = 1	19.3	83.71	1	1	12.57	yes	
[UCMA_MOUSE]	Ucma	Unique cartilage matrix-associated protein OS = Mus musculus GN = Ucma PE = 1 SV = 1	16.6	76.49	1	1	9.42	yes	yes
[SAP_MOUSE]	Psap	Prosaposin OS = Mus musculus GN = Psap PE = 1 SV = 2	61.4	69.91	3	1	3.59	yes	yes
[CCD80_MOUSE]	Ccdc80	Coiled-coil domain-containing protein 80 OS = Mus musculus GN = Ccdc80 PE = 1 SV = 2	107.5	60.21	1	1	1.26	yes	
[DAG1_MOUSE]	Dag1	Dystroglycan OS = Mus musculus GN = Dag1 PE = 1 SV = 4	96.8	57.62	1	1	1.79		yes
[CRTAP_MOUSE]	Crtap	Cartilage-associated protein OS = Mus musculus GN = Crtap PE = 1 SV = 3	46.1	51.66	1	1	2.75	yes	
[CDSN_MOUSE]	Cdsn	Corneodesmosin OS = Mus musculus GN = Cdsn PE = 2 SV = 2	54.3	36.81	1	1	3.21		yes

*Identified in bone-conditioned media Bone-CM1, Bone-CM2, or both as indicated.

### Biochemical analysis of secreted factors in Calvarial-CM

**Figure 3:**
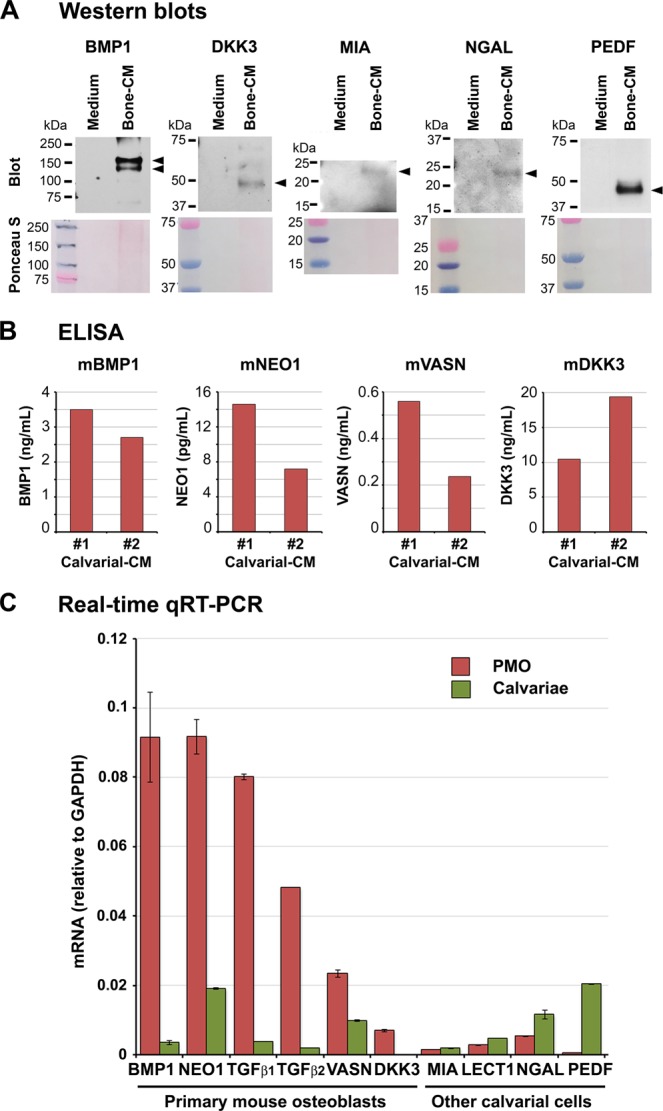
Expression of bone-secreted proteins in the calvariae. (**A**) Western blots. A 20X concentrated BSA-free Calvarial-CM (Bone-CM) together with a 20X concentrated control BGJb medium were incubated with antibodies against bone-secreted factors as indicated. Original Western blots are shown in Supplemental Fig. S1. (**B**) ELISA. Protein levels of select bone-secreted factors in two independent preparations of mouse Calvarial-CM, Calvarial-CM #1 and Calvarial-CM #2, were analyzed by ELISA. m, mouse. (**C**) Real-time qRT-PCR. Total RNAs were prepared from PMO versus total calvariae and analyzed by qRT-PCR. mRNA levels normalized against GAPDH controls were compared between PMO versus calvariae. A higher PMO to calvariae ratio indicates that the protein is expressed by osteoblasts. Error bars represent triplicate determinations.

We used a 20X concentrated BSA-free Calvarial-CM (Bone-CM) for Western blot analysis and examined the expression of five secreted proteins, i.e., BMP1 (110 kDa), DKK3 (38 kDa), MIA (15 kDa), NGAL (25 kDa), and PEDF (50 kDa). The predicted molecular weight of each protein is indicated in parenthesis. We detected NGAL and PEDF with molecular mass of 25 kDa and 50 kDa, respectively, as predicted, in Bone-CM (Fig. 3A). Western blot for BMP1 showed protein bands at 150 kDa and 110 kDa. Because BMP1 contains 5 potential N-link glycosylation sites (UniProt database), these results indicate that BMP1 in Bone-CM is likely glycosylated. DKK3 appeared as a major band at 50 kDa and a minor band at 75 kDa. As DKK3 also contains 4 potential N-link glycosylation sites (UniProt database), DKK3 in Bone-CM may also be glycosylated. MIA is a small molecular size protein that contains two disulfide bonds (UniProt database). We found that MIA also appeared slightly higher than predicted in three independent blots (see Supplemental Fig. S1), the basis of which is currently unclear. These bands were not detected in control BGJb medium (Fig. 3A), thus confirming the presence of these secreted factors in Bone-CM. Using ELISA, we further determined the concentrations of several bone-secreted factors in two independent mouse Calvarial-CM preparations, Calvarial-CM #1 and Calvarial-CM #2. We found that the Calvarial-CM #1 and #2 contains 2.7 and 3.5 ng/mL BMP1, 7.2 and 14.6 pg/mL NEO1, 0.2 and 0.6 ng/mL VASN and 10.5 and 19.4 ng/mL DKK3, respectively (Fig. 3B). The concentrations of these factors in Calvarial-CM are likely much lower than those in physiological or pathological conditions *in vivo* because in the experimental condition, the bone-secreted factors are secreted into the culture media and are vastly diluted. In contrast, PCa cells may encounter bone-secreted factors in much higher concentrations than the levels we observed by ELISA in the Calvarial-CM because PCa cells are found to be adjacent to the tumor-induced bone^36^ in the *in vivo* bone microenvironment. Together, the Western blot and ELISA analyses confirm the presence of candidate bone-secreted factors in Calvarial-CM.

### Expression of secreted factors from different types of cells in calvariae

The newborn mouse calvariae contain osteoblasts as well as osteoclasts, osteocytes and fibroblasts. We next determined whether the candidate bone-secreted factors identified by MS/MS are mainly expressed in mouse osteoblasts or other cell types within the calvariae. Primary mouse osteoblasts (PMOs) were isolated from newborn mouse calvariae by digestion with collagenase plus trypsin as described in Materials and Methods (see Fig. 1A). RNA was prepared from PMOs or total calvariae. The expression of the candidate bone-secreted factors in PMOs or calvariae was analyzed by quantitative RT-PCR using mouse-specific primers (Table 3). A higher PMO/calvariae mRNA ratio indicates that a factor is likely to be expressed by osteoblasts. Using this criteria, we found that neogenin, vasorin and DKK3 are mainly expressed in PMOs, while MIA, LECT1 and NGAL are mainly expressed in other cells in the calvariae (Fig. 3C). We also analyzed the expression of two known matrisome-associated ECM regulator proteins that have important functions in bone: BMP1, a metalloproteinase that regulates the formation of the bone matrix^37,38^, and pigment epithelium-derived factor (PEDF), a potent antiangiogenesis factor^39^ that was also shown to promote osteoblast differentiation and mineralization of the bone matrix (Table 1)^40^. We found that BMP1 is mainly expressed in PMOs while PEDF is expressed by other cells in the calvariae (Fig. 3C). As controls for known osteoblast-expressed proteins, we showed that two TGFβ family members TGFβ1 and TGFβ2 are expressed by PMOs. TGFβ2 was previously shown to be a dormancy-inducing factor while TGFβ1 was not^3,14^. TGFβ1 also did not affect cellular dormancy in HNSCC HEp3 cells^14^, breast cancer cells^20^ or PCa cells^3^.

### Dormancy-inducing activity of bone-secreted factors

**Figure 4:**
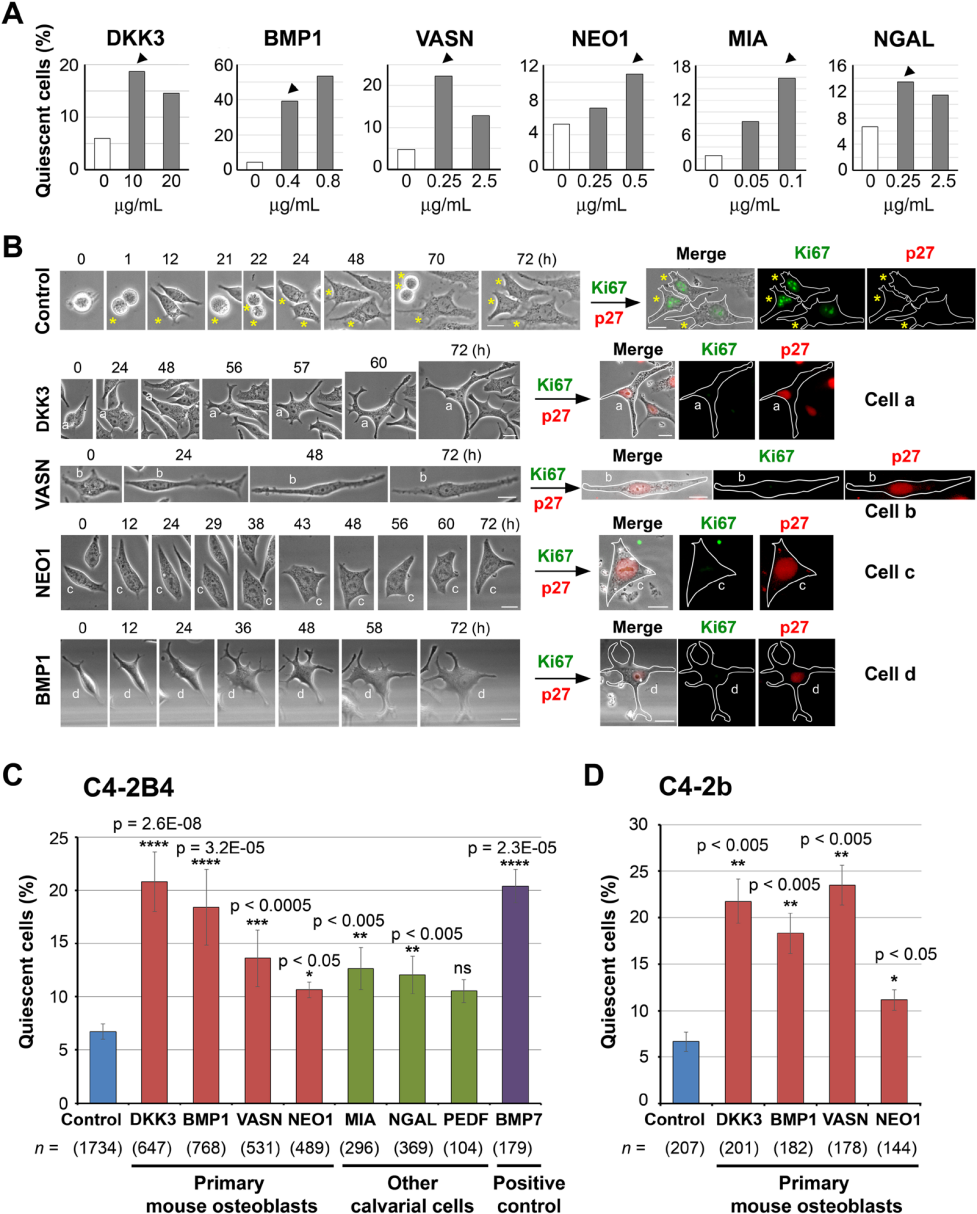
Bone-secreted factors induce cellular quiescence in PCa cells. (**A**) Dose response. C4-2B4 PCa cells were treated without or with various recombinant human bone-secreted factors at different concentrations and analyzed by live-cell imaging as in Fig. 1. About 100 cells were monitored for each factor at each concentration. Using this approach, we obtained an empirically-derived optimal concentration to be used for each factor (arrowheads): DKK3 (10 µg/mL), BMP1 (0.4 µg/mL), VASN (0.25 µg/mL), NEO1 (0.5 µg/mL), MIA (0.1 µg/mL), and NGAL (0.25 µg/mL). (**B**) (Left) Phase contrast brightfield images. Cells were plated in a Q4 glass-bottom dish, treated with and without various recombinant proteins, and analyzed by live-cell imaging. Representative images are shown for control cells and cells treated with recombinant human DKK3 (cell a), vasorin (cell b), neogenin (cell c), and BMP1 (cell d). Asterisks (*) follow a control cell through 3 cell divisions. Round cells are cells undergoing mitosis. (Right) Immunofluorescence images. At the end of live-cell imaging, cells were immediately fixed and co-immnostained for Ki67 (proliferation marker) and p27 (dormancy marker), and merged with phase contrast images. Cell outlines are traced for ease of view. All bars, 20 µm. (**C**) C4-2B4 cells were treated with various bone-secreted factors, using concentrations as determined in (A), and analyzed by live-cell imaging. PEDF was used at 0.25 µg/ml^47^. The dormancy factor BMP7 (0.4 µg/ml)^50^ was used as a positive control. % quiescent cells that did not divide relative to total cells counted were quantified (mean ± s.e.m), except for PEDF and BMP7 (mean ± s.d.). *n*, number of cells monitored. N, number of independent experiments for control (N = 24), DKK3 (N = 10), BMP1 (N = 10), vasorin (N = 9), neogenin (N = 4), MIA (N = 4), NGAL (N = 6), PEDF (N = 2), BMP7 (N = 2). P values were by *t* test. ns, not significant. (**D**) C4-2b cells were treated with various bone-secreted factors as indicated, monitored by live-cell imaging, and analyzed as in (**C**). *n*, number of cells monitored. N, number of independent experiments for control (N = 3), DKK3 (N = 3), BMP1 (N = 3), vasorin (N = 2), neogenin (N = 2). P values were by *t* test.

To examine the potential dormancy-inducing activity of candidate bone-secreted factors, recombinant proteins corresponding to select factors were first screened for their ability to induce cellular quiescence in PCa cells. We conducted a dose response analysis for each selected bone-secreted factors. We used factor concentrations reported in the literature^41–47^ and tested several concentrations to ensure that the dosages used do not cause toxicity, i.e., cell death, in PCa cells using live-cell imaging. Based on the response, we selected the following concentrations for further study: DKK3 (10 µg/mL), BMP1 (0.4 µg/mL), vasorin (0.25 µg/mL), neogenin (0.5 µg/mL), MIA (0.1 µg/mL), or NGAL (0.25 µg/mL) (Fig. 4A, arrowheads). Live-cell imaging showed that there was an increase in non-dividing quiescent cells when C4-2B4 cells were incubated with recombinant human DKK3, vasorin, neogenin or BMP1 (Fig. 4B, left), as illustrated by examples of cells that exhibited a “dormant” phenotype after treatment with DKK3 (cell a), vasorin (cell b), neogenin (cell c), or BMP1 (cell d). In contrast, the majority of control C4-2B4 PCa cells underwent several rounds of cell division, as illustrated by following one cell from F0 (T = 0 h) as it divided into F1 (T = 1 h), F2 (T = 22 h) and then F3 (T = 70 h) progenies (Fig. 4B, asterisks). Of note, although these non-proliferating quiescent cells did not divide, they exhibited a low level of movement with cell shape changes during time-lapse recording (Fig. 4B, left).

We further confirmed the dormancy status of the non-dividing quiescent cells by co-immunostaining for Ki67 and p27. Proliferating cells express the proliferation marker Ki67 in all phases of the cell cycle (G1, S, G2, M) but not during quiescence (G0). In quiescent breast cancer cells, the cell cycle inhibitor p27 has been shown to be upregulated and translocated into the nucleus^48,49^. Thus, cellular dormancy is characterized by Ki67 negativity and p27 positivity. Control cells that underwent several rounds of cell division over 72 h were found to be Ki67 positive and p27 negative (Fig. 4B, right). In contrast, quiescent cells a – d that did not divide upon treatment with DKK3, vasorin, neogenin or BMP1 were Ki67 negative and showed nuclear translocation of p27 (Fig. 4B, right). The combination of these morphological, cell cycle and biochemical analyses suggests that DKK3, vasorin, neogenin and BMP1 induced cell cycle arrest leading to cellular dormancy in C4-2B4 cells.

The levels of dormancy induced by the select bone-factors were quantified directly by cell counting. By combining 72 independent live-cell imaging experiments, around 1,700 control cells and 3,500 cells treated with various recombinant factors were monitored at the single cell level (Fig. 4C). Amongst the selected factors, we found that DKK3 significantly increased the level of quiescent, non-dividing C4-2B4 cells by about 3-fold, to 20% when compared to 7% in untreated control cells (Fig. 4C). This level of DKK3-mediated dormancy induction was comparable to that observed with the dormancy-inducing factor BMP7^50^, which was used as a positive control. Two other factors, vasorin and neogenin, were also effective in inducing cellular quiescence, though to a lesser extent than DKK3. Of the three factors expressed by other calvarial cells, MIA and NGAL exerted a modest effect on cellular quiescence on C4-2B4 cells while PEDF effects did not reach stastistical significance relative to control cells. BMP1, which differs from BMP2 – BMP16 in that it is not a TGFβ-like protein, but is important for the formation and mineralization of bone^38,51^, was found to induce a significant 18% of C4-2B4 cells to become quiescent. These analyses show that a variety of bone-secreted factors display different extent of dormancy-inducing activity on C4-2B4 cells.

We tested the dormancy-inducing activity of bone-secreted factors on another PCa cell line C4-2b^52^. Although C4-2b and C4-2B4 cells are both sublines derived from LNCaP cells^53,54^, we previously observed that these cells show differences in cell morphology and motility under the same culture conditions^3^. That C4-2b and C4-2B4 cells have distinct properties is supported by recent reports that these cells exhibit differences in their epithelial-to-mesenchymal transition state^55^. We found that DKK3, BMP1 and vasorin induced significant cellular quiescence to 18–24% relative to 7% in control untreated C4-2b cells (Fig. 4D), while neogenin’s effects were modest. Taken together, these results show that multiple factors secreted by different cell types within bone can induce cellular dormancy in PCa cells.

### Phospho-p38MAPK in dormancy signaling

**Figure 5:**
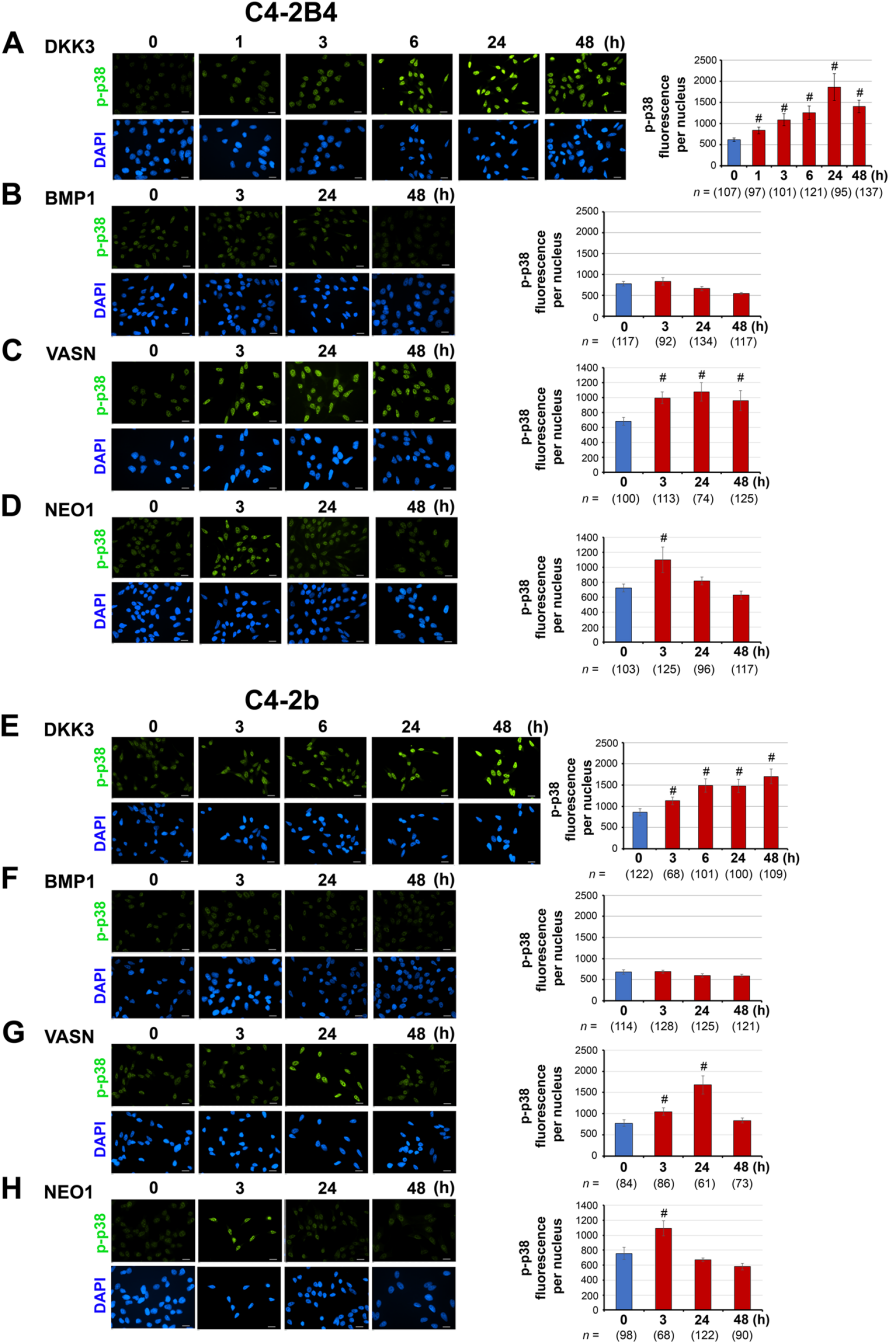
Dormancy signaling via phospho-p38MAPK. C4-2B4 (**A**–**D**) and C4-2b (**E**–**H**) cells were incubated with select bone-secreted factors over a time course for up to 48 h as indicated. Cells at different time points were immunostained for phospho-p38MAPK (p-p38) and counterstained with DAPI for DNA. Representative images of p-p38 nuclear translocation are shown. The levels of p-p38 fluorescence signals per nucleus were quantified using NIS-Elements software. All bars, 20 µm. *n*, number of nuclei analyzed. P values were by *t* test. ^#^p < 0.0001.

Activation of p38MAPK through phosphorylation is one of the major signaling pathways utilized by dormancy-inducing factors^14,56^. We examined whether these newly-identified dormancy factors induce dormancy through p38MAPK phosphorylation^3^. Immunofluorescence imaging followed by quantification showed that phospho-p38MAPK (p-p38) became significantly enriched in the nucleus of C4-2B4 cells in response to DKK3 (Fig. 5A), vasorin (Fig. 5C) and neogenin (Fig. 5D) stimulation, indicating that these factors induced p38MAPK activation. Both DKK3 and vasorin stimulated p-p38 nuclear translocation within 3 h with sustained activation maintained up to 24–48 h in C4-2B4 cells (Fig. 5A,C), while neogenin stimulated a transient p-p38 nuclear translocation with a maximum at 3 h followed by a decline to basal levels by 48 h (Fig. 5D). Interestingly, although BMP1 activated dormancy induction (Fig. 4C), BMP1 did not stimulate p-p38 nuclear translocation in C4-2B4 cells (Fig. 5B), suggesting that an alternative signaling pathway for dormancy induction is used by BMP1. A similar pattern of p-p38 nuclear translocation in response to DKK3 (Fig. 5E), vasorin (Fig. 5G), and neogenin (Fig. 5H) was observed in C4-2b cells. As also observed in C4-2B4 cells, BMP1 did not stimulate p-p38 nuclear translocation in C4-2b cells (Fig. 5F), although BMP1 was effective in stimulating dormancy induction in C4-2b cells (Fig. 4D). These results suggest that DKK3, vasorin and neogenin activated canonical p-p38MAPK dormancy signaling, albeit with differences in the kinetics of p38MAPK activation, and BMP1 employs an alternative signaling pathway to dormancy induction.

### Effect of dominant-negative p38αMAPK mutant on dormancy-inducing activity of bone-secreted factors

**Figure 6:**
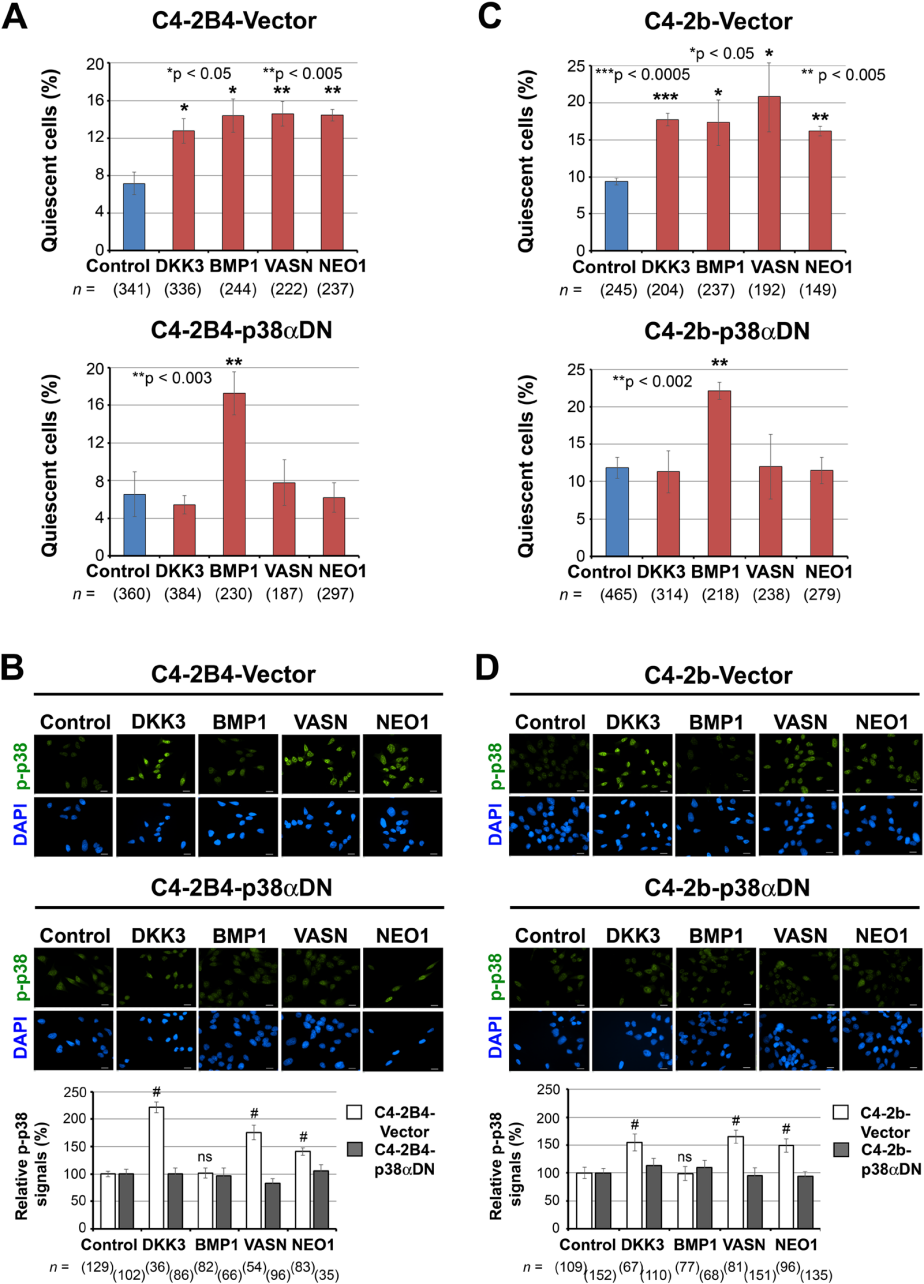
Dominant-negative p38αMAPK prevents dormancy induction *in vitro* for DKK3, VASN and NEO1, but not BMP1. (**A**) C4-2B4 cells stably-expressing empty vector or p38αDN mutant were treated with the indicated factors for 72 h and analyzed by live-cell imaging. Quantification of % quiescent cells that did not divide over 72 h relative to total cells examined (mean ± s.e.m.). *n*, number of cells monitored. N, number of independent experiments for C4-2B4-Vector cells: control (N = 4), DKK3 (N = 4), BMP1 (N = 3), vasorin (N = 3), neogenin (N = 3); and for C4-2B4-p38αDN cells: control (N = 5), DKK3 (N = 6), BMP1 (N = 3), vasorin (N = 3), neogenin (N = 3). P values were by *t* test. (**B**) Cells in (**A**) were treated with the indicated factors for 3 h and immunostained for p-p38 (upper and middle). All bars, 20 µm. Relative p-p38 signals in the nucleus of factor-treated cells compared to those in control cells (lower). *n*, number of nuclei analyzed. P values were by *t* test. ^#^p < 0.0001; ns, not significant. (**C**) C4-2b-Vector or C4-2b-p38αDN cells were treated as in (**A**), and % cellular quiescence was analyzed by live-cell imaging. *n*, number of cells monitored. N, number of independent experiments for C4-2b-Vector cells: control (N = 3), DKK3 (N = 3), BMP1 (N = 3), vasorin (N = 3), neogenin (N = 2); and for C4-2b-p38αDN cells: control (N = 6), DKK3 (N = 5), BMP1 (N = 3), vasorin (N = 3), neogenin (N = 3). P values were by *t* test. (**D**) Cells in (**C**) were treated as in (**B**) and relative p-p38 signals in the nucleus were analyzed as in (**B**).

We next examined whether activation of p38MAPK plays a role in mediating the dormancy-inducing activity of bone-secreted factors, using PCa cells transduced with a dominant-negative p38α MAPK (p38αDN) mutant construct^3^. DKK3, vasorin and neogenin were unable to stimulate dormancy induction in C4-2B4-p38αDN cells (Fig. 6A, lower), while exhibiting dormancy induction in the control C4-2B4-vector cells (Fig. 6A, upper), suggesting that overexpression of p38αDN effectively blocked dormancy induction by these factors. These results are consistent with an increase in p-p38 nuclear translocation in response to these factors in C4-2B4-vector control cells (Fig. 6B, upper and lower) but not in C4-2B4-p38αDN cells (Fig. 6B, middle and lower). In contrast, BMP1 continued to elicit dormancy activation in both C4-2B4-Vector control cells (Fig. 6A, upper) as well as C4-2B4-p38αDN cells (Fig. 6A, lower), in agreement with a lack of p-p38 nuclear translocation (Fig. 6B, middle and lower) in response to BMP1. Similar results were observed when C4-2b cells overexpressing p38αDN were examined. Dormancy induction by DKK3, vasorin and neogenin was prevented in C4-2b-p38αDN cells (Fig. 6C, lower), but not in C4-2b-Vector control cells (Fig. 6D, upper). That BMP1 does not signal through p38MAPK was observed by the lack of p-p38 nuclear translocation in C4-2b-Vector control cells (Fig. 6D) and continued dormancy induction in C4-2b-p38αDN cells in the presence of BMP1 (Fig. 6C, lower). These results further confirm that BMP1 mediates dormancy induction independently of p38MAPK. Thus, activation of p38MAPK signaling is required to mediate the dormancy-inducing activity of DKK3, vasorin and neogenin, but not BMP1, in PCa cells.

## Discussion

We have identified a unique set of bone-secreted factors that can induce cellular dormancy in PCa cells. These factors belong to different protein families. While many of these factors are found to be expressed by osteoblasts, some are expressed by other stromal cells in the calvariae. Most of these factors induce cellular dormancy in PCa cells through activation of the p38MAPK signalling pathway, albeit with different kinetics of p38MAPK activation. Interestingly, BMP1-induced cellular dormancy is mediated through a p38MAPK-independent pathway. Thus, dormancy induction from the bone microenvironment involves factors secreted from multiple cell types with various signal transduction mechanisms. Such variations may result in varied dormancy duration seen in the clinical setting. These observations may contribute to the future development of strategies for the prevention or treatment of bone metastasis.

The transforming growth factor-β (TGFβ) family proteins are the first group of proteins shown to exhibit dormancy-inducing activity^57^. These proteins include TGFβ2^3,14^, GDF10 (BMP-3b)^3^, and BMP7^50^. Other factors that exhibit dormancy-inducing activities include GAS6^9,10^, thrombospondin (TSP-1)^20^, Wnt5a^8^ and LIF^58^. The panel of bone-secreted factors identified in our studies expands this growing family of dormancy-inducing factors. One of the secreted factors is DKK3, a divergent member of the DKK family of secreted Wnt signaling antagonists^59^. Overexpression of DKK3 has been shown to inhibit prostate tumor growth and metastasis by limiting TGFβ/Smad signaling^24,25,60^. DKK3 was also found to be downregulated in a range of human cancers^59,61^. Interestingly, DKK3 does not seem to act as a Wnt signaling antagonist^59,62^. Recent studies in a bone fracture and repair model using a DKK3-GFP reporter mouse suggest that DKK3 is expressed in the mesenchymal progenitor cells in the periosteum and in the bone marrow and is required for cartilage cell development by blocking osteogenesis^63^. Our studies revealed a potential role for DKK3 as a dormancy-inducing factor that signals through p38MAPK to modulate PCa growth in bone. Vasorin, another bone-secreted factor, is a type I single-pass transmembrane protein^35^ that contains tandem leucine-rich repeats, an epidermal growth factor (EGF)-like motif, and a fibronectin type III-like motif in the extracellular domain, which are motifs involved in protein-protein interactions. It has been shown that the extracellular domain of vasorin can be cleaved into a soluble ectodomain that binds directly to TGFβ1 and antagonizes TGFβ1 signaling^35,64^. Although our study showed that vasorin induces PCa cell dormancy through p38MAPK *in vitro*, whether antagonizing TGFβ1 signaling contributes in part to how vasorin induces cellular quiescence of PCa cells is unknown. Another dormancy-inducing factor is BMP1. The ECM-regulator BMP1 is a member of the BMP1/Tolloid-like family of metalloproteinase and is found in many matrisomes (Table 1). In bone, BMP1 is highly expressed in areas of bone matrix formation, and mutations in BMP1 result in osteogenesis imperfecta or “brittle bone disease” in humans^51^. BMP1 is reported to affect BMP/TGFβ pathways indirectly through the release of latent binding proteins that tether BMP2/4 as well as TGFβ1-3 in the ECM matrix^38,65^. Our studies showed that BMP1 contributes to dormancy in PCa cells through a p38MAPK-independent pathway. Whether BMP1’s metalloproteinase activity is involved in its dormancy-inducing activity is not known.

We note that the empirically-determined concentrations of recombinant factors used in our studies are about 1,000 times higher than those found in Calvarial-CM. As PCa cells are found adjacent to the tumor-induced bone^36^, it is likely that PCa cells may encounter bone-secreted factors in higher concentrations *in vivo*. Indeed, the high factor concentrations used in our studies are within the range reported in the literature. As an example, similar concentrations of human DKK3 at 10–50 µg/mL were used to block the proliferation of human kidney KPK1 cancer cells^41^; human BMP1-3, an alternatively-spliced variant of BMP1, at 150–1000 ng/mL was observed to increase the expression of p21, a cell cycle inhibitor, in human embryonic kidney 293 (HEK293) cells^42^; human MIA at 50 ng/mL was used to inhibit the invasive activity of melanoma cells^45^; and PEDF at 250 ng/mL was used to induce osteoblastic differentiation of human mesenchymal stem cells^47^.

We found that different cell types, besides osteoblasts, within the tumor microenvironment are also involved in the induction of tumor cell dormancy. That bone marrow stromal cells secrete factors to induce cellular dormancy has been previously reported. Kobayashi *et al*.^50^ showed that HS5 bone marrow stromal cells secrete BMP7 that inhibits PC3 PCa cell proliferation. We also confirmed the dormancy-inducing activity of BMP7 on C4-2B4 PCa cells (Fig. 4C), but found that BMP7 is not expressed by either osteoblasts or other calvarial cells (data not shown). Ghajar *et al*.^20^ showed that endothelial cells in mature vessels secrete TSP-1 that leads to breast tumor cell dormancy. Price *et al*.^66^ showed that dormant breast cancer cells are tethered to the bone marrow vascular niche through C-X-C chemokine receptor type 4 (CXCR4) interactions with stromal cell-derived factor (SDF-1) that is enriched in the perisinusoidal vascular region. Thus, various dormancy-inducing factors secreted by different cell types within the tumor microenvironment are involved in the induction of tumor cell dormancy.

We have previously performed proteomics analysis of osteoblast exosomes, which we termed osteosomes, isolated from undifferentiated/proliferating versus differentiated/mineralizing primary mouse osteoblasts^67^. It is possible that the proteomics analysis of Calvarial-CM contains both bone-secreted soluble factors and proteins derived from osteosomes. We compared the 132 secreted proteins identified in Bone-CM (Table 1) with the 373 proteins previously identified in osteosomes^67^. We found that 25 proteins (18.9%) in the Bone-CM were also found in osteosomes (Supplemental Table S3). These include the cytokine MIF; chemokines S10A9, S10AB and S10A6; ECM glycoproteins TENN, AEBP1 and LAMB1; and ECM regulator proteins SERPH, MMP13, CD109, CATB and A2MP (Supplemental Table S3). Notably, the bone-secreted factors DKK3, BMP1, vasorin, neogenin, MIA and NGAL that exhibited dormancy-inducing activity in PCa cells are not found in the osteosomes. Whether osteosomal proteins can mediate dormancy induction in PCa cells remains to be determined.

Only a limited number of signaling pathways that mediate dormancy responses have been reported. A change in mitogenic signaling from the Ras-induced extracellular signal-regulated kinase (ERK) pathway to the p38MAPK pathway has been shown to lead to a switch from proliferation to cellular dormancy^68–70^. Our studies together with reports by others showed that the p38MAPK signaling pathway is involved in PCa cell quiescence mediated by TGFβ2, GDF10, BMP7, DKK3, vasorin and neogenin (Figs. 5 and 6)^3,14,50^. Interestingly, we note that factors that elicited a more sustained p-p38MAPK activation and nuclear translocation for up to 24–48 h, such as DKK3 and vasorin, generated a higher level of dormancy induction than those that generated a transient p-p38MAPK response, such as neogenin, in PCa cells (Fig. 5). While the significance of the different kinetics of p38MAPK activation is currently unclear, they may have effects on the duration of the dormancy state. How p38MAPK activation imparts specificity to dormancy induction is also unknown. In our previous study, we found that p38MAPK phosphorylates RB at the novel N-terminal S249/T252 sites^71^ to block PCa cell proliferation, a process that leads to an increase in the cell cycle CDK inhibitor p27, followed by G0-G1 cell cycle arrest and cellular quiescence^3^. While p38MAPK represents a canonical dormancy signaling pathway, other pathways that signal to cellular dormancy also exist. Johnson *et al*.^58^ reported that the leukemia inhibiting factor LIF, a member of the IL-6 cytokine family, induces breast cancer cell dormancy by activating STAT3 signaling molecules. Notably, we found that BMP-1 induces cellular dormancy through a p38MAPK-independent pathway (Fig. 6), which remains to be identified.

How dormancy-inducing factors regulate the dormancy gene network is not clear. Aguirre-Ghiso^72^ and Segall’s^73^ groups have reported that p38MAPK signaling reprograms tumor cells to acquire a quiescence program through regulating a network of transcription factors and target genes (“dormancy signature genes”) whose expression promotes quiescence of metastatic head and neck squamous cell carcinoma HEp3 cells and breast cancer cells, respectively. These genes constitute a dormancy transcription network that confers cellular dormancy. The transcription factors include NR2F1/COUPTF1^73,74^, BHLHE41/Dec2/Sharp-1^14,73^ and FOXM1^72,73^, which may serve as a “switch” for converting active tumor cells into “dormant” cells. BHLHE41 has been shown to suppress the metastasis of aggressive triple-negative breast cancers through the degradation of hypoxia inducing factor HIF that promotes tumor angiogenesis^75,76^. How p38MAPK signaling leads to BHLHE41 gene expression and which dormancy signature genes might be regulated by BHLHE41 in PCa cells remain to be elucidated.

Delineating the mechanisms that lead to exit from dormancy is important for preventing tumor relapse. Similar to dormancy entry, dormancy exit can be due to intrinsic properties of the tumor cells that overcome dormancy and/or extrinsic signals encountered in the tumor microenvironment^77,78^. An increase in the expression of the BMP inhibitor Coco has been shown to render highly metastatic MDA-MB-231 breast cancer cells non-dormant^79^ as well as to promote metastatic outgrowth of 4TO7 mammary tumor cells in lung by inhibit-ing BMP signaling, thus allowing the reactivation of tumor cells from dormancy^79^. Increased expression of VCAM1 in mammary tumor cells has been shown to induce bone lysis, resulting in the release of growth factors from bone that enable tumor cells to exit dormancy^4,80^. Increased signaling through β1 integrin was shown to induce both D2A1 breast cancer cells^48^ and LuCaP PDX PCa cells^81^ to transition from quiescence to proliferation. Additionally, alterations in the tumor microenvironment as a result of hormonal changes, aging or therapies^77,78^ may lead to a loss of dormancy-inducing factors. Together, these observations suggest that entry into and exit out of cellular dormancy may involve distinct pathways.

Currently, therapy strategies that target dormant tumor cells, which are resistant to chemotherapies^82,83^, are limited^77,83^. It seems that keeping dormant tumor cells dormant indefinitely or eradicating dormant tumor cells while they are dormant would be safer than reactivation followed by chemotherapy. In this context, maintaining the dormancy-conferring microenvironment would be an important aspect of the prevention strategy. Identification of mechanisms that support tumor cell survival during prolonged periods of dormancy may elucidate strategies to target dormant tumor cells. Because dormant tumor cells are responsible for cancer relapse, further understanding of tumor cell dormancy mechanisms is warranted.

## Materials and Methods

### Cell lines, reagents and antibodies

Human PCa C4-2B4 (gift from Robert Sikes, University of Delaware)^53,54^ and C4-2b (gift from Leland Chung, Cedars-Sinai Medical Center)^52^ were used. C4-2B4 and C4-2b cells transduced with either pBMN-I-GFP Vector alone or retroviral-expressing dominant-negative p38αMAPK (p38αDN) mutant, containing mutations (Thr180-Gly-Tyr182 to Ala180-Gly-Phe182) in the activation loop between the two kinase domains^84^, were as previously described^3^. The identity of all cell lines was verified by polymorphic Short Tandem Repeat loci (STR) profiling, and all cell lines are mycoplasma free. Human recombinant proteins DKK3, BMP1, Neogenin (R&D); Vasorin (Novoprotein); MIA (Peprotech); NGAL, PEDF (SinoBiologicals); and BMP7 (Prospec) were obtained from commercial sources. Antibodies for Ki67 (Dako #MIB-1), p27Kip1 (Cell Signaling #D69C12), phospho-p38MAPK (Thr180/Tyr182) (Cell Signaling #4511), DKK3 (Proteintech #10355-1-AP), BMP1 (abcam #ab205394), MIA (Santa Cruz #sc-37735), NGAL (Santa Cruz #sc-515876), and PEDF (R&D #AF-1177) were obtained from commercial sources.

### Conditioned media from mouse calvariae (Calvarial-CM) and primary mouse osteoblast preparation

Calvariae were prepared from 20 2–5 day-old pups and incubated in BGJb medium, a chemically-defined media designed for the growth of fetal rat long bones (Thermo Fisher), and supplemented with 0.1% BSA as previously described^12^. Conditioned media from two independent batches of calvariae were prepared at 48 h (Calvarial-CM #1 and Calvarial-CM #2) and used for dormancy and ELISA assays. To prepare conditioned media for mass spectrometry analysis, calvariae were incubated in BSA-free BGJb medium for 48 h (Bone-CM). Two independent Bone-CM1 and Bone-CM2 were prepared. Primary mouse osteoblasts (PMOs) were prepared as follows. Calvariae from ~35 new born pups were digested in α-MEM containing 0.1 mg/ml collagenase P and 0.25% trypsin^11^. The first two digestions (15 min each) that contained fibroblasts and other undefined cell types were discarded. After the third digestion (37 °C for 1 h), supernatant enriched in PMOs together with the calvariae pieces were plated in α-MEM supplemented with 10% FBS for 3 to 4 days. The calvariae pieces were then removed and discarded. The cultures were then trypsinized and the PMOs were plated on 10 cm plates. All animal experiments in this work were conducted in accordance with NIH guidelines and have been approved by the institutional review board at the U. Texas M. D. Anderson Cancer Center.

### Identification of proteins in bone-conditioned media by liquid chromatography-tandem mass spectrometry (LC-MS/MS)

The Bone-CM samples were concentrated 20-fold in Centricon-10 (Millipore). The concentrated samples were precipitated by acetone (acetone/sample 5:1) and placed at −20 °C overnight. The precipitated proteins were resuspended in 10 µL of Rapigest (2 mg/mL in 100 mM ammonium bicarbonate) (Waters) plus 30 µL of 50 mM ammonium bicarbonate, and denatured by heating to 100 °C for 10 min. The denatured samples were cooled to room temperature and digested with 200–400 ng sequencing-grade trypsin (20 ng/μL in 0.02% formic acid) (Promega) at 37 °C overnight. The digested samples were dried down using Speedvac and reconstituted in 1% formic acid. The resulting peptides were analyzed by liquid chromatography–tandem mass spectrometry (LC–MS/MS) on an Orbitrap Elite mass spectrometer (Thermo Scientific) as previously described^67^. HPLC analyses were performed with Dionex Ultimate 3000. Samples were injected into a Phenomenex core–shell C18 DB column (2.7 μm 15 cm), with mobile phase compositions of A: 0.1% formic acid in water and B: 0.1% formic acid in acetonitrile and with a flow rate of 100 µL/min. The gradient was held isocratic at 2% B for 2 min, ramped up to 35% at 105 min, ramped up to 80% at 106 min, maintained at 80% until 110 min, ramped down to 2% at 110.1 min, and held at 2% until 120 min.

The MS parameters and scan strategy were: (a) mass range for MS1: 400–1300; (b) mass resolution for MS1: 500 000; (c) mass window for precursor ion selection: 0.5 d; (d) number or precursors selected for tandem MS in each scan cycle: maximum in 2 s; (e) mass analyzer for tandem-MS: MS1: Orbitrap; MS2: Iontrap; (f) charge state screening parameters: 2–4; (g) relative collision energy: 30%; (h) dynamic exclusion settings: 15 s.

Data processing of the MS results were as follows: (a) Database: SwissProt_2018_02 *Mus* database (17 024 sequences), Total sequences: 556 825, Total residues: 199 652 254; (b) Search engine: Mascot 2.6 via Proteome Discoverer 1.4; (c) Precursor and product ion mass tolerances: Peptide Mass Tolerance: 10 ppm, Fragment Mass Tolerance: 0.8 Da; Use average precursor mass: False; (d) Peptide scoring options: Peptide Cut Off Score: 10, Peptide Without Protein Cut Off Score: 5; (e) Protein scoring options: MudPIT Scoring: False, Protein Relevance Threshold: 20, Protein Relevance Factor: 1; (f) Enzyme specificity: Trypsin, 2 missed cleavages allowed; (g) Dynamic modifications: Oxidation (M), Gln → pyro-Glu (N-term Q), Trioxidation (C); (h) Method for false discovery rate (FDR) assessment: Processing node 7: Target Decoy PSM Validator; Decoy Database Search: Target FDR (strict): 0.01.

### Western blot analysis

A 20X concentrated preparation of BSA-free mouse Calvarial-CM (Bone-CM) along with a 20X concentrated control BGJb medium were analyzed by Western blot. NuPAGE MES SDS running buffer (ThermoFisher) was used to better resolve small to medium-size proteins. After gel transfer, the filters were cut into two pieces at either the 25 or 37 kDa mark to accommodate the concurrent analysis of bone-secreted factors with large and small molecular sizes. The original Western blots are shown in Supplemental Fig. S1.

### ELISA

Protein levels of bone-secreted factors in two independent mouse Calvarial-CM preparations were analyzed by ELISA as previously described^12^. ELISA kits (mouse) were from AVIVA System Biology for mBMP1 (OKCD02401) and mDKK3 (OKCD02498), and from MyBioSource for mNEO1 (MBS2533529) and mVasorin (MBS2706258).

### Quantitative real-time RT-PCR (qRT-PCR)

Total RNA was prepared from PMO or calvariae and analyzed by quantitative RT-PCR as previously described^12^, to examine the expression of bone-secreted factors. Mouse-specific primers are as listed (Table 3).

**Table 3:** PCR primers used for bone-secreted factors.

Mouse BMP1-Forward	AGTTTGGCATCGTGGTCCAT
Mouse BMP1-Reverse	TACTCCTGCCCTGGCTGTAT
Mouse BMP7-Forward	CGTCCAGACACTGGTTCACTT
Mouse BMP7-Reverse	GAGGACAGAGATGGCGTTGA
Mouse DKK3-Forward	TTGTTCATTCGAATTGGGCGG
Mouse DKK3-Reverse	ACACAGCAAAATACCCCCGA
Mouse LECT1-Forward	TTACCACCAGCAGGAAGGAG
Mouse LECT1-Reverse	GTAGCCTCCCAGTGGTTCAC
Mouse MIA-Forward	GTCCATGATGGTGTGGTCCC
Mouse MIA-Reverse	TGGAGATAGGATGGCTGCATTC
Mouse NGAL-Forward	ATGCACAGGTATCCTCAGGT
Mouse NGAL-Reverse	TGGCGAACTGGTTGTAGTCC
Mouse NEO1-Forward	CTCACACAGTGCCAGATCCC
Mouse NEO1-Reverse	ATGTTGGTCTTCCACCGGAC
Mouse PEDF-Forward	CTACAGAACCCCCAGAGGGA
Mouse PEDF-Reverse	AAGCAGAGCCCGTTCATGTT
Mouse TGFβ1-Forward	AGCTGCGCTTGCAGAGATTA
Mouse TGFβ1-Reverse	AGCCCTGTATTCCGTCTCCT
Mouse TGFβ2-Forward	AATGGCTCTCCTTCGACGTG
Mouse TGFβ2-Reverse	AGGTGCCATCAATACCTGCAA
Mouse VASN-Forward	GAGGTGAAGGACTGAGGCCC
Mouse VASN-Reverse	CTTCTGTCCCAGGAGACGACTG
Human/mouse GAPDH-Forward	CCCAGAAGACTGTGGATG
Human/mouse GAPDH-Reverse	GCAGGGATGATGTTCTGG

### Live-cell time-lapse imaging

PCa cells were plated in Hi-Q4 glass-bottom dishes (Ibidi) and cultured in RPMI-1640 containing 10% FBS. Cells were washed with RPMI-1640 containing 0.1% FBS before the addition of Calvarial-CM or recombinant human proteins. Concentrations used for the various recombinant human proteins were empirically derived. Images (20X objective) were acquired every 20 min for 72 h in a BioStation (Nikon) as previously described^3^. Data were compiled using NIS-Elements (Nikon) software.

### Immunofluorescence imaging

Immediately following live-cell recording, cells were fixed and permeabilized, incubated with anti-Ki67 mouse monoclonal antibody (1:1000) or co-incubated with anti-Ki67 and anti-p27 rabbit monoclonal antibody (1:700) overnight, followed by goat-anti-mouse IgG-Alexa Fluor 488 and goat-anti-rabbit IgG-Texas Red (ThermoFisher) secondary antibodies for 3 h, and re-imaged on the BioStation^3^. PCa cells were also incubated with various bone-secreted factors over a time course, followed by immunostaining with phospho-p38MAPK (1:200) antibody overnight and counter-staining with DAPI for DNA. Images were acquired on a TE2000 widefield microscope system (Nikon, Lewisville, TX). The level of phospho-p38MAPK fluorescence per nucleus was quantified using NIS-Elements 5.11.01 (Nikon) software.

### Statistical analysis

Data quantification was performed by using the Student’s *t*-test and expressed as mean ± s.e.m. *P* values of <0.05 were considered statistically significant.

## Supplementary information

Supplemental Figure S1

Supplemental Table S1

Supplemental Table S2

Supplemental Table S3
