# Supplementary material for: Bone secreted factors induce cellular quiescence in prostate cancer cells
Source: Sci Rep. 2019 Dec 9;9:18635. doi: 10.1038/s41598-019-54566-4 (PMC6901558; doi:10.1038/s41598-019-54566-4)
Supplement: Supplementary file 1 — Supplemental Figure S1 [file 41598_2019_54566_MOESM1_ESM.docx]

**­**

**Bone secreted factors induce cellular quiescence in prostate cancer cells**

Li-Yuan Yu-Lee, Yu-Chen Lee, Jing Pan, Song-Chang Lin, Tianhong Pan, Guoyu Yu, David H. Hawke, Bih-Fang Pan, Sue-Hwa Lin


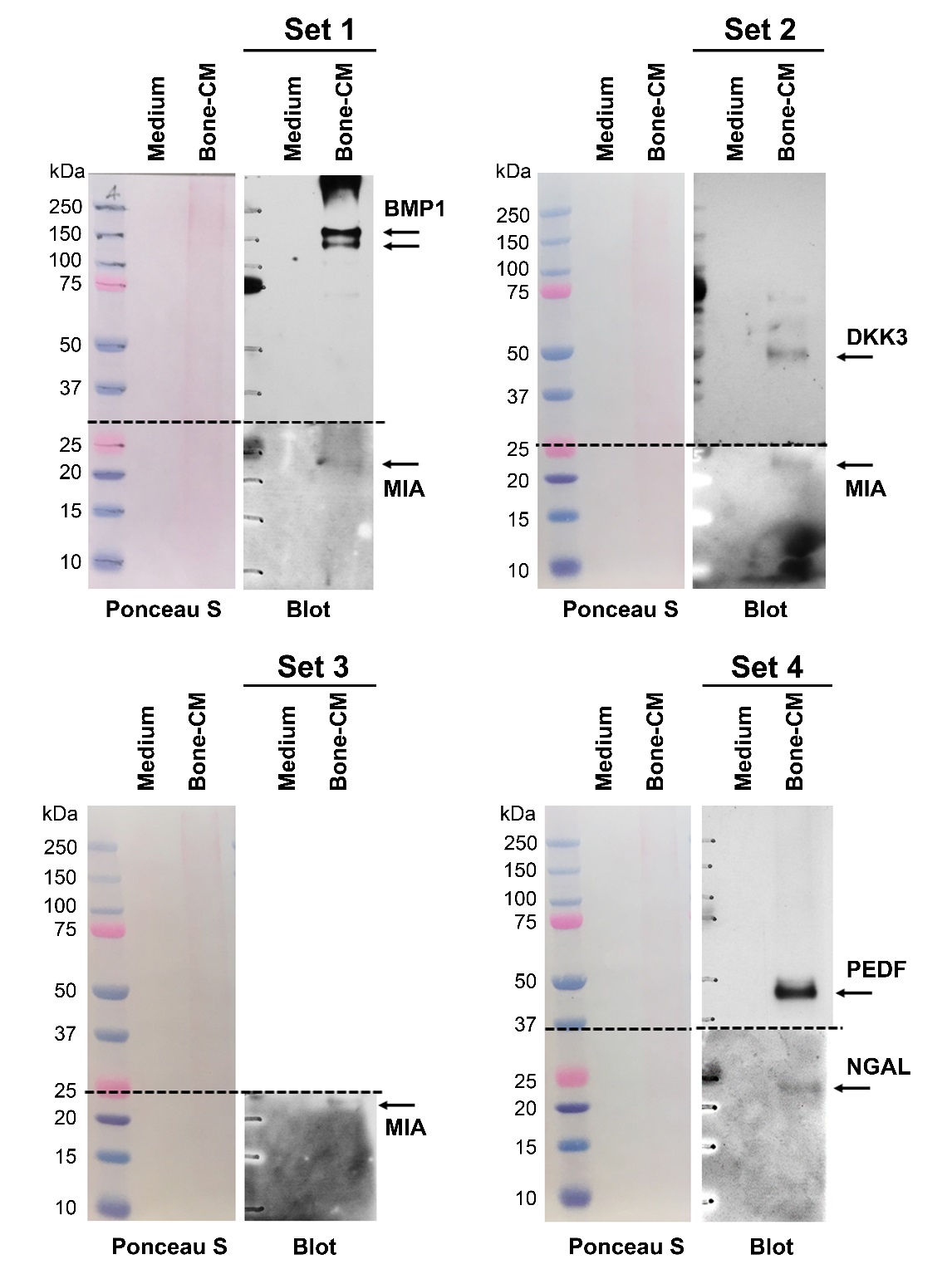


**Supplemental Figure S1.** Original Western blots for data presented in Fig. 3A.

A 20X concentrated preparation of BSA-free mouse Calvarial-CM (Bone-CM) along with a 20X concentrated control BGJb medium were analyzed by Western blot. Four sets of samples were run on SDS-PAGE. NuPAGE MES SDS running buffer was used to better resolve small to medium-size proteins. The filters were cut along the dotted lines into two pieces at either the 25 or 37 kDa mark to accommodate the concurrent analysis of bone-secreted factors with large and small molecular sizes. Set 1 filter was used to detect BMP1 and MIA; Set 2 for DKK3 and MIA; Set 3 for MIA; and Set 4 for PEDF and NGAL. Note, MIA was analyzed in three independent blots. MIA in Set 2 was shown in Fig. 3A.
